# Nanoscale transformations of amphiboles within human alveolar epithelial cells

**DOI:** 10.1038/s41598-022-05802-x

**Published:** 2022-02-02

**Authors:** Ruggero Vigliaturo, Maja Jamnik, Goran Dražić, Marjetka Podobnik, Magda Tušek Žnidarič, Giancarlo Della Ventura, Günther J. Redhammer, Nada Žnidaršič, Simon Caserman, Reto Gieré

**Affiliations:** 1 Department of Earth and Environmental Science, University of Pennsylvania, Philadelphia, USA; 2 Department of Molecular Biology and Nanobiotechnology, National Institute of Chemistry, Ljubljana, Slovenia; 3 Department of Materials Chemistry, National Institute of Chemistry, Ljubljana, Slovenia; 4 Department of Biotechnology and System Biology, National Institute of Biology, Ljubljana, Slovenia; 5 Department of Geological Sciences, University of Roma Tre, Rome, Italy; 6 INFN-Istituto Nazionale Di Fisica Nucleare, Frascati (Rome), Rome, Italy; 7 INGV, Via di Vigna Murata 605, 00143 Rome, Italy; 8 Department of Materials Science and Physics, University of Salzburg, 5020 Salzburg, Austria; 9 Department of Biology, Biotechnical Faculty, University of Ljubljana, Ljubljana, Slovenia; 10 Center of Excellence in Environmental Toxicology, University of Pennsylvania, Philadelphia, USA

**Keywords:** Biogeochemistry, Natural hazards, Environmental sciences, Environmental chemistry

## Abstract

Amphibole asbestos is related to lung fibrosis and several types of lung tumors. The disease-triggering mechanisms still challenge our diagnostic capabilities and are still far from being fully understood. The literature focuses primarily on the role and formation of asbestos bodies in lung tissues, but there is a distinct lack of studies on amphibole particles that have been internalized by alveolar epithelial cells (AECs). These internalized particles may directly interact with the cell nucleus and the organelles, exerting a synergistic action with asbestos bodies (AB) from a different location. Here we document the near-atomic- to nano-scale transformations induced by, and taking place within, AECs of three distinct amphiboles (anthophyllite, grunerite, “amosite”) with different Fe-content and morphologic features. We show that: (i) an Fe-rich layer is formed on the internalized particles, (ii) particle grain boundaries are transformed abiotically by the internal chemical environment of AECs and/or by a biologically induced mineralization mechanism, (iii) the Fe-rich material produced on the particle surface does not contain large amounts of P, in stark contrast to extracellular ABs, and (iv) the iron in the Fe-rich layer is derived from the particle itself. Internalized particles and ABs follow two distinct formation mechanisms reaching different physicochemical end-states.

## Introduction

Exposure to amphibole asbestos is in many cases associated with asbestosis, pleural abnormalities, bronchogenic carcinomas and mesotheliomas^1^. The carcinogenic potency of amphibole asbestos was proven both epidemiologically and toxicologically^2^. Asbestos-related tumors are difficult to diagnose and have long latency periods, and it is not clear whether there is a minimum threshold exposure for carcinogenesis^3^. Moreover, the disease-triggering mechanism of these minerals is still puzzling^4^, although new findings have recently advanced the understanding of asbestos-related mesothelioma, lung cancer, and fibrosis^1,5,6^. To support the medical community in tracking the fate of elongate mineral particles (EMPs) in cells (EMP is a term that includes asbestos minerals^7^), we have approached the problem from the opposite end: instead of focusing on the amphibole-related transformation of cells, we studied how amphibole particles internalized by human lung cells are transformed at the near-atomic- to nano-scale.

Asbestos is an all-inclusive, confusing, non-scientific, industrial term^8–12^. Moreover, the related malignancies can also be caused by non-regulated asbestiform minerals^13^, with an unclear role of nano-sized EMPs, non-regulated fibers, cleavage fragments and split mineral particles internalized by human lung cells^14–20^. For these reasons, our study focuses on the in-vitro uptake by alveolar epithelial cells (AECs) and the transformation of amosite (the asbestiform variety of the mineral grunerite) and non-asbestiform amphiboles (anthophyllite and grunerite). AEC injury is a key trigger, which promotes the development of asbestosis and lung cancer^1,21–24^. Here, we tested the interaction of the three selected minerals with the AEC line A549 (ATCC® CCL­185™), to study the alterations induced by the AECs. Our systematic approach can be more effective in reducing and partially controlling the involved variables (e.g., chemical composition and surface physicochemical state) than the frequently used comparison between crystal-chemically and physically dissimilar minerals like chrysotile *vs*. amphibole asbestos and/or asbestiform zeolites.

This study focuses on the intracellular transformation of amphibole particles, and complements the extensive literature describing asbestos bodies (ABs), i.e., extracellular Fe-covered “fibers” found in lung tissue, and their role in triggering related malignancies. Once internalized, these amphibole particles may act as a source of reactive oxygen species (ROS)^25^ in close contact with nuclei and cell organelles, and induce DNA damage in concert with the “external” action and stimuli triggered by asbestos (and ABs)^14–20^, and the systemic response of the body to their presence. Asbestos bodies have been characterized in detail^26–29^, also in terms of Fe-valence state^30^. Our study, on the other hand, documents in unprecedented detail the transformation of micro- and nano-sized EMPs within AECs, using a unique set of transmission electron microscopy (TEM) techniques (Table 1). It thus provides unmatched information on the physicochemical end-states of internalized EMPs, which so far have not been reported in the literature. These details may be crucial for the interpretation of biological, toxicological, and potentially carcinogenic properties and effects, which have so far not yet been determined so exhaustively for particles internalized by cells^18^. The results show that both composition and structure of the amphibole particles modified within AECs differ considerably from those of extracellular ABs, which have been described in some detail in the literature. We further observed differences between transformations occurring in abiotic experiments and our in-vitro experiments, and we hypothesize that they can be related to the so-called “vital effect”.

**Table 1:** Summary of the S/TEM techniques used for the characterization of both the starting and the interacted material.

Technique^1^	Feature/property	Collected data for systematic analyses on the starting material
acSTEM BF-MAADF, acHRTEM	Shape/morphology	Qualitative observation on a minimum of 500 particles
SAED, acHRTEM and acSTEM BF-MAADF	Crystal structure and crystallinity/amorphization	Observation of 50 SAED patterns and 100 HR images (50 in TEM and 50 in STEM mode)
acSTEM-EDXS	Chemical composition	Recording of mapping areas to obtain the chemical composition of a minimum of 100 particles (dwell time 2 ms per pixel)
acSTEM Dual-EELS	Fe-oxidation state in the crystal structure	Recording of a minimum of 20 EELS spectra from areas of 25 × 25 nm (3 frames for 10 s exposure in the core-loss region)

^1^Additional Abbreviations: acSTEM = aberration corrected Scanning Transmission Electron Microscopy; BF = Bright Field; MAADF = Medium-Angle Annular Dark Field; SAED = Selected Area Electron Diffraction; HR = High-Resolution; EDXS = Energy-Dispersive X-Ray Spectroscopy; EELS = Electron Energy-Loss Spectroscopy.

## Results

### The amphibole particles before the experiments

As mentioned above, the amphibole samples were selected such as to expose the AECs to minerals with different features, in particular their chemical composition and shapes. Specifically, grunerite (Gru) and amosite (Amo) have a similar Fe content, significantly higher than that of anthophyllite (Ath; see the detailed characterization in the Supplementary Information S.1 and S.2). In terms of morphological features, most of the Amo particles can be classified as asbestos, whereas both Gru and Ath particles are better categorized as cleavage fragments and/or as mostly non-asbestiform amphiboles.

**Figure 1:**
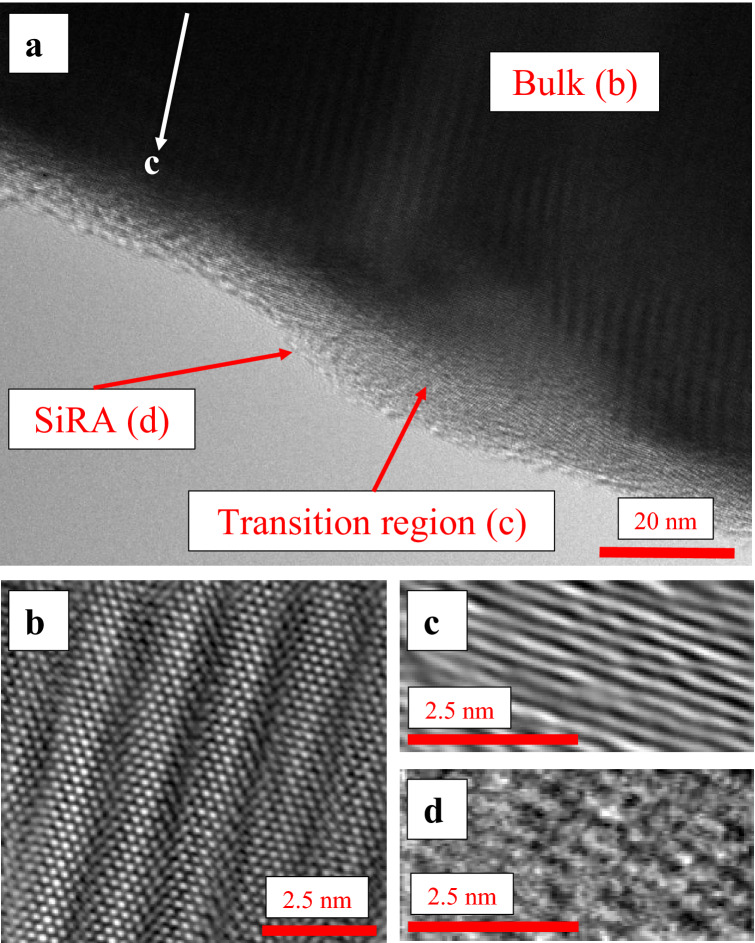
High-resolution TEM images of an amosite particle boundary (**a**) Overview of the amosite particle boundary showing the areas detailed in (**b**), (**c**) and (**d**); (**b**) TEM-Inverse Fast Fourier Transform (IFFT) image showing the bulk of the amosite particle, which is crystalline at the atomic scale; (**c**) Transition region between the crystalline amosite core and the Si-rich amorphous surface layer (SiRA); it can be identified by the loss of one of the symmetries; (**d**) SiRA.

Combination of HRTEM and BF acSTEM (for abbreviations, see Table 1) revealed that the bulk structures of all three amphiboles are highly crystalline (Fig. 1a,b; see also Supplementary Information S.2). However, near the surface of all three minerals there is a transition zone in which the crystallinity is partially lost (Fig. 1c). This transition zone is in turn covered by a Si-rich amorphous layer (SiRA) of variable extent and thickness (Fig. 1a,d), in which the Si framework is rearranged (Fig. 1d) and other cations (e.g., Mg^2+^ and Ca^2+^) are depleted (See Supplementary Information S.2.3), as previously described by Germine and Puffer^31^ in tremolite-actinolite EMPs extracted from the lungs of miners in Quebec (Canada).

In some cases, regardless of the amphibole species, the SiRA is partially covered by an irregular amorphous Fe-rich layer^32,33^. The presence of a discontinuous, oxidized, Fe-rich layer is also reflected by the high Fe-valence state determined by Dual-EELS (see below) in this region.

### Transformation of amphibole particles within AECs

**Figure 2:**
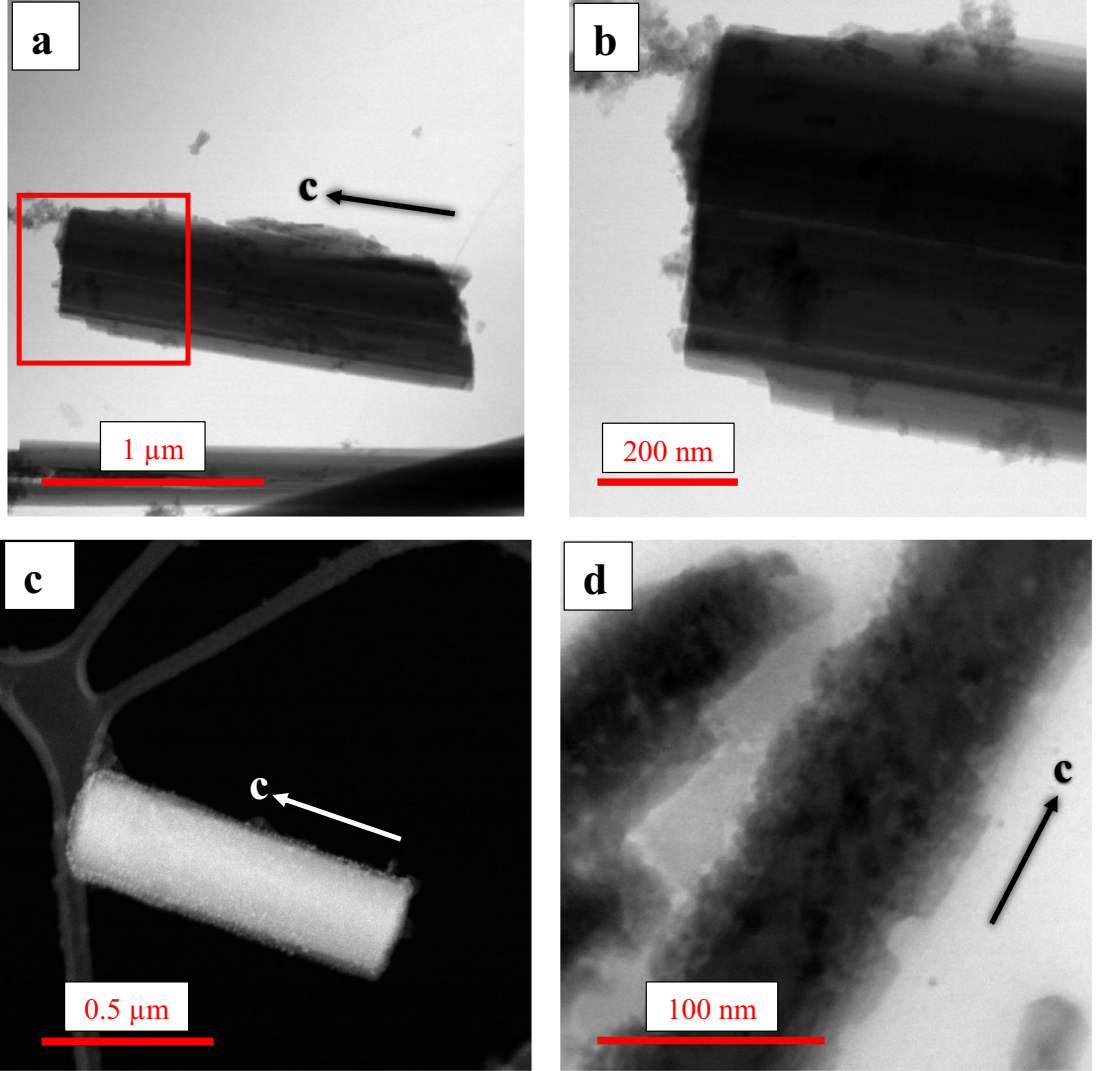
TEM images of amphiboles after interaction with AECs. (**a**) A nearly non-modified cleavage fragment of amosite; (**b**) Detail of the image shown in (**a**) (red square), in which stepped lamellar boundaries, rounded corners and streaking along the *c*-axis can be recognized; (**c**) acSTEM-HAADF image of an anthophyllite particle after interaction; the particle is completely covered by an Fe-rich layer containing several Fe-rich nanoparticles, which are visible as bright spots; (**d**) BF image of elongated particles of grunerite after interaction; particles display rough, highly modified grain boundaries, compromised morphology and clear signs of dissolution of the amorphous layer.

The particles retrieved from within AECs after the experiments displayed two types of appearances, regardless of their mineralogical identity: type-1 particles with only limited evidence of dissolution (Fig. 2a,b); these are characterized by a valence state of Fe near the surface that is statistically identical, or slightly reduced, compared to that at the grain boundaries of the starting material; and type-2 amphibole particles with pronounced modifications at their surface (Fig. 2c,d), showing formation of Fe-rich clusters and Fe-rich nanoparticles, and oxidation of a surficial Fe-rich amorphous layer (additional particles with different habits are shown in Supplementary S.3, Fig. S.3.1).

Type-1 particles are very similar to naturally weathered amphiboles in terms of chemical composition and physical transformations (Table 2, Supplementary Information Fig. S.3.1c,d, and S.3.2a,b)^31–33^: both show modified grain boundaries and both lack Fe-rich clusters and nanoparticles in the Fe-rich layers at their surface. However, the Fe-rich layer in naturally weathered amphiboles tends to be less extended, and less thick than that observed for type-1 particles extracted from the AECs (e.g., Supplementary Information Fig. S.2.2.1, and Fig. S.3.2a,b). Most of the Amo particles retrieved from the AECs belong to this typology (Fig. 2a,b). The extracted Gru particles were observed to belong equally to types 1 and 2, whereas the extracted Ath particles were most extensively transformed and largely belong to type 2.

**Table 2:** Oxide composition (in wt%) of the amphibole particles before (starting material) and after (retrieved from cells) the interaction with AECs. Data obtained by acSTEM-EDXS bulk analyses. Before the interaction: *n* = 100 for each of the minerals; after the interaction: *n* = 19, 24 and 59 for anthophyllite, grunerite and amosite, respectively.

		Mean	σ_n-1_	Max	Min		Mean	σ_n-1_	Max	Min	Relative difference (in percentages)
**Anthophyllite**											
**Before Interaction**	Na_2_O	N.D	–	N.D	N.D	**After Interaction**	N.D	–	N.D	N.D	–
	MgO	22.87	1.19	24.43	21.94		7.62	0.68	8.10	7.14	−66.68
	Al_2_O_3_	6.03	1.00	6.77	4.63		1.61	0.64	2.06	1.15	−73.30
	SiO_2_	59.47	1.96	61.14	56.68		43.22	0.10	43.29	43.15	−27.33
	K_2_O	0.01	0.01	0.02	N.D		N.D	−	N.D	N.D	−100
	CaO	0.17	0.08	0.28	0.09		N.D	−	N.D	N.D	−100
	TiO_2_	N.D	–	N.D	N.D		N.D	–	N.D	N.D	–
	MnO	0.28	0.03	0.31	0.25		N.D	–	N.D	N.D	−100
	FeO	11.25	1.65	13.59	10.03		47.56	1.22	48.42	46.69	+ 322.76
	Mg/(Mg + Fe_tot_)	0.61					0.11				−81.97
**Grunerite**											
**Before Interaction**	Na_2_O	N.D	–	N.D	N.D	**After Interaction**	N.D	–	N.D	N.D	–
	MgO	7.87	1.45	8.81	5.38		10.09	0.63	10.88	9.33	+ 28.21
	Al_2_O_3_	0.99	0.25	1.19	0.56		0.94	0.63	1.32	N.D	−5.05
	SiO_2_	50.05	1.16	51.33	48.46		55.90	1.23	56.73	54.09	+ 11.69
	K_2_O	N.D	–	N.D	N.D		N.D	–	N.D	N.D	–
	CaO	0.12	0.07	0.25	0.06		0.08	0.06	0.12	N.D	−33.33
	TiO_2_	N.D	–	N.D	N.D		N.D	–	N.D	N.D	–
	MnO	N.D	–	N.D	N.D		N.D	–	N.D	N.D	–
	FeO	40.97	2.07	43.51	38.70		32.99	1.95	35.32	30.97	−19.48
	Mg/(Mg + Fe_tot_)	0.13					0.19				+ 46.15
**Amosite**											
**Before interaction**	Na_2_O	0.01	0.02	0.03	N.D	**After interaction**	N.D	–	N.D	N.D	−100
	MgO	8.83	0.45	9.15	8.17		6.97	0.02	6.98	6.95	−21.07
	Al_2_O_3_	0.82	0.39	1.26	0.45		1.02	0.23	1.18	0.86	−24.39
	SiO_2_	54.60	2.01	56.90	52.00		45.79	5.76	49.86	41.72	−16.14
	K_2_O	0.01	0.02	0.04	N.D		N.D	–	N.D	N.D	−100
	CaO	0.11	0.05	0.19	0.08		0.04	0.01	0.04	0.03	−63.64
	TiO_2_	N.D	–	N.D	N.D		N.D	–	N.D	N.D	–
	MnO	0.32	0.03	0.36	0.29		N.D	–	N.D	N.D	−100
	FeO	35.31	2.10	38.21	33.29		46.20	5.55	50.12	42.27	+ 30.84
	Mg/(Mg + Fe_tot_)	0.16					0.10				−37.5

N.D = not determined

**Figure 3:**
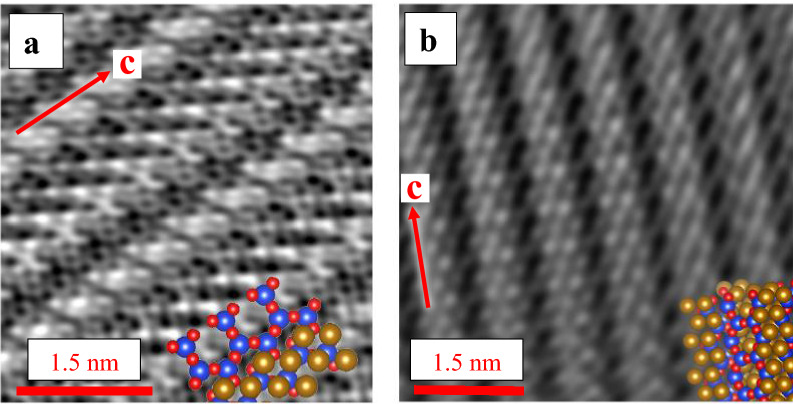
Two different orientations of the crystalline core region of amosite with superimposed crystal structure, where the blue, red and orange atoms represent Si, O, and Fe, respectively. AcSTEM images showing (**a**) a crystal *before* the interaction with AECs; and (**b**) a crystal (in a different orientation) *after* 48 h of interaction with AECs.

In both typologies, however, the bulk is highly crystalline, as documented down to the near-atomic scale, in the starting material as well as after interaction with the AECs (Fig. 3).

The mean Mg/(Mg + Fe) value, determined by acSTEM-EDXS, did not change significantly in the bulk Gru and Amo particles during their interaction with the AECs, but decreased substantially in the case of Ath (Table 2). The distinct change in the Ath composition is mainly due to a loss of Mg, Al and Si, which caused a relative increase in the concentration of Fe (Table 2).

The external amorphous material (most likely a mix of SiRA and Fe-rich material) is also visible after the interaction between particles and AECs, but it is usually thicker and has a more irregular, rough topography in comparison to the starting material (Supplementary Information S.3.2). At the grain boundary of all three amphibole types, we observed an Fe-rich layer lying on top of the SiRA. This Fe-rich layer is generally less developed in type-1 than in type-2 particles. The valence state of Fe at the particle boundary (25 × 25 nm square) of the interacted amphiboles is statistically identical, or slightly lower, when compared to that at the grain boundaries of the starting material (Table 3).

**Table 3:** Valence state determined by EELS at particle grain boundaries (25 × 25 nm) before and after the interaction with AECs.

		Before interaction		After interaction	
**Anthophyllite**	Average valence state	2.72	(*n* = 35)	2.56	(*n* = 8)
	σ_n-1_	0.14		0.07	
	Maximum	3.12		2.67	
	Minimum	2.46		2.44	
**Grunerite**	Average valence state	2.43	(*n* = 26)	2.08	(n = 10)
	σ_n-1_	0.26		0.03	
	Maximum	2.96		2.13	
	Minimum	2.06		2.02	
**Amosite**	Average valence state	2.44	(*n* = 38)	2.28	(*n* = 13)
	σ_n-1_	0.31		0.10	
	Maximum	3.10		2.49	
	Minimum	2.09		2.18	

**Figure 4:**
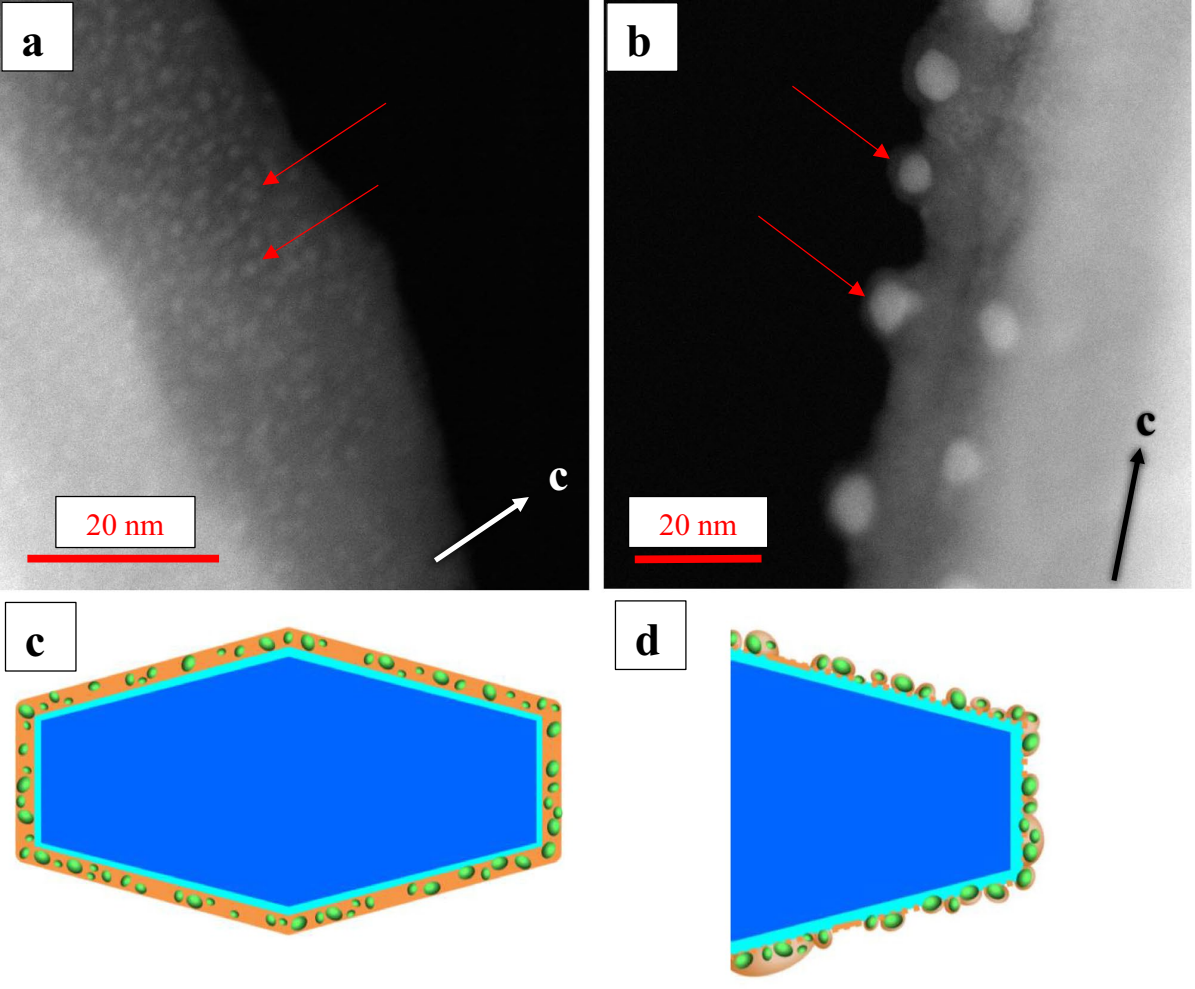
acSTEM-MAADF images of (**a**) visible Fe-clusters, appearing in light grey (examples highlighted with red arrows), embedded in a Fe-rich layer covering a grunerite particle extracted from AECs (white part of the image on the left); (**b**) amorphous Fe-rich nanoparticles (examples highlighted with red arrows) surrounded by Fe-rich material covering an anthophyllite particle (white part on right side of image) extracted from AECs. The two diagrams (**c**,**d**) show simplified cross-section models of an amphibole particle after its interaction with the AECs. (**c**) Crystalline amphibole (dark blue, representing the “bulk”), which is completely covered by the SiRA (light blue) and an outermost Fe-rich amorphous layer (orange) with embedded amorphous Fe-rich nanoparticles; (**d**) Crystalline amphibole whose Fe-rich layer (light orange) is discontinuous and heterogeneously covers the underlying SiRA.

The Fe-rich layer is generally amorphous (Fig. 4a,b) and either occurs as a continuous envelope around the amphibole particles (Fig. 4a,c), or it only partially covers the SiRA (Fig. 2d, Supplementary Information Fig. S.3.2.b). When well developed (i.e., in type-2 particles), the Fe-rich layer can be decorated by clusters of Fe^2+^ (Fig. 4a), which seem to develop into amorphous Fe-rich nanoparticles (Fig. 4, and Supplementary Information S.3, Fig. S.3.2c,d). Here, we define as “clusters” Fe-rich regions of rounded or ellipsoidal shape that do not have a clear or distinguishable interface with the surrounding Fe-rich layer but are visible mostly because of their MAADF contrast (e.g., Fig. 4a); their diameter is smaller than 2 nm (measured along the larger axis cutting through the cluster). In contrast, the “nanoparticles” have a distinct boundary, which clearly separates them from the surrounding Fe-rich layer (e.g., Fig. 4b); the shape of the nanoparticles is more easily distinguishable even without the use of MAADF (e.g., Supplementary Fig. S.3.2c, d). The average dimensions of the Fe-rich clusters and nanoparticles are 1.24 ± 0.21 nm (46 measurements) and 7.44 ± 2.30 nm (84 measurements), respectively (Table 4).

**Table 4:** Diameter range and statistical information for the Fe-rich clusters and nanoparticles occurring in the surficial amorphous, Fe-rich layer on amphiboles.

	Minimum	Maximum	Mean	σ_n-1_	*n*
Cluster diameter (nm)	0.93	1.93	1.24	0.21	46
Nanoparticle diameter (nm)	3.37	14.58	7.44	2.30	84

Both Fe-rich clusters and nanoparticles are characteristic of the highly modified type-2 particles, and rarely occur in type-1 particles, implying that they are generated at the grain boundary of the amphibole crystallites *inside* the AECs. Each individual Fe-rich cluster or Fe-rich nanoparticle is always embedded in the amorphous Fe-rich layer and never lies on the surface of the amphibole (Fig. 4, and Supplementary Fig. S.3.2).

**Figure 5:**
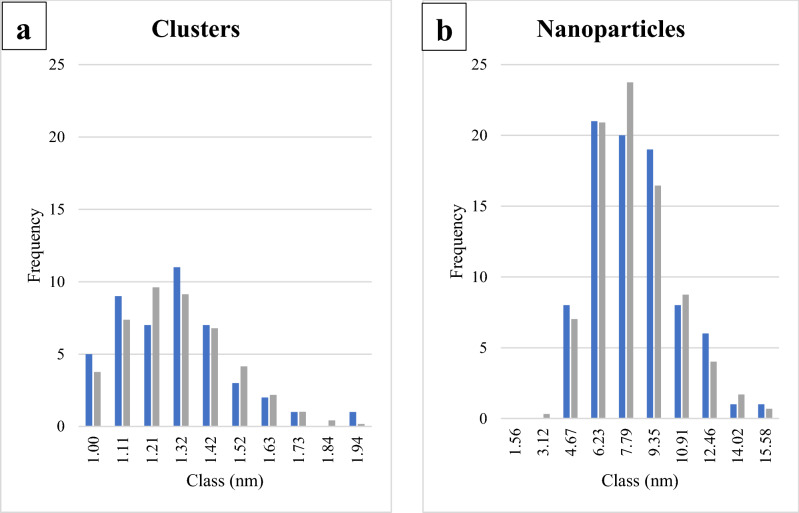
Experimentally determined (blue) and calculated (log-normal fits; grey) size distributions of clusters and nanoparticles occurring in the amorphous Fe-rich layer on amphibole particles extracted from AECs, indicating an abiogenic or BIM process of particle formation. (**a**) size distribution of clusters on a grunerite particle; (**b**) size distribution of nanoparticles on an anthophyllite particle. The x-axis labels show the upper limit of each bin (10 bins total for each chart).

The log-normal dimensional distributions of the Fe-rich clusters (Null hypothesis (H_0_) = log-normal distribution; Kolmogorov–Smirnov test: D = 0.050; p-value = 0.977; α = 0.05; Chi-square test: chi-square (observed value) = 2.916; chi-square (critical value) = 14.067; GDL = 7; p-value = 0.893; α = 0.05) and nanoparticles (Null hypothesis (H_0_) = log-normal distribution; Kolmogorov–Smirnov test: D = 0.060; p-value = 0.993; α = 0.05; Chi-square test: chi-square (observed value) = 6.851; chi-square (critical value) = 14.067; GDL = 7; p-value = 0.445; α = 0.05) (Fig. 5) are consistent with abiogenic and/or biologically induced mineralization (BIM), but not with biologically controlled mineralization (BCM) processes (BCM would exhibit a negatively skewed size distribution)^34–37^. Briefly, during BIM, the precipitation of minerals occurs as a consequence of the interaction between the biological activity and the environment, whereas during BCM, the subject organism directly controls the nucleation and growth, the habit, and the location of the mineral through is cellular activity^34–37^.The dimensions of the clusters (Table 4, Fig. 5a) were measured on a completely visible Fe-rich layer covering a Gru particle (e.g., Fig. 4a), whereas those of the nanoparticles (Fig. 5b) were determined on a completely visible Fe-rich layer covering an Ath particle (e.g., Fig. 4b). Even though this method of using size distributions to determine the nature of mineralization processes has never been applied to minerals that interacted with, or were generated by eukaryotic cells, our findings are compatible with the literature data on abiotic amphibole dissolution in simulated biofluids^38–41^. We believe that this method could be applied to nanoparticle generation in eukaryotic systems following further testing.

We used Dual-EELS to highlight differences in the Fe-valence state among the observed Fe-rich objects (clusters, nanoparticles, and amorphous layer) in the modified surface layers of all analyzed amphiboles retrieved from the AECs (Table 5). Even though the number of analyses is relatively small for each type of amphibole, the difference in Fe-valence state is significant between the clusters and the Fe-rich layer, whereby the Fe-rich layer is in all cases more oxidized than the clusters. This observation also holds when comparing the individual objects across all types of amphiboles: overall, the Fe-valence state in all analyzed Fe-rich clusters (*n* = 21) is 2.05 ± 0.05, whereas that in the surrounding Fe-rich layer (*n* = 12) is 2.36 ± 0.11 (Table 5). Furthermore, this dataset confirms that the highest number of Fe-rich nanoparticles (*n* = 16) was observed on Ath particles, which, in comparison to Gru and Amo, were most strongly modified chemically during the interaction with the AECs (Table 5).

**Table 5:** Average Fe-valence state of Fe-rich clusters, nanoparticles, and amorphous layer grown during the interaction with AECs, as determined by acSTEM Dual-EELS investigations.

		**Fe-rich clusters**	**Fe-rich nanoparticles**	**Fe-rich layer**
**Ath**		***n* = 9**	***n* = 16**	***n* = 5**
	**Mean**	2.05	2.19	2.35
	**σ**_**n-1**_	0.05	0.11	0.16
	**Maximum**	2.15	2.45	2.55
	**Minimum**	2.00	2.01	2.14
**Gru**		***n* = 11**	***n* = 2**	***n* = 1**
	**Mean**	2.04	2.21	2.31
	**σ**_**n-1**_	0.04	0.20	-
	**Maximum**	2.14	2.35	2.31
	**Minimum**	2.00	2.07	2.31
**Amo**		***n* = 1**	***n* = 2**	***n* = 6**
	**Mean**	2.16	2.25	2.37
	**σ**_**n-1**_	–	0.06	0.07
	**Maximum**	2.16	2.29	2.46
	**Minimum**	2.16	2.21	2.29
**Overall**		***n* = 21**	***n* = 20**	***n* = 12**
	**Mean**	2.05	2.20	2.36
	**σ**_**n-1**_	0.05	0.11	0.11
	**Maximum**	2.16	2.45	2.55
	**Minimum**	2.00	2.01	2.14

**Figure 6:**
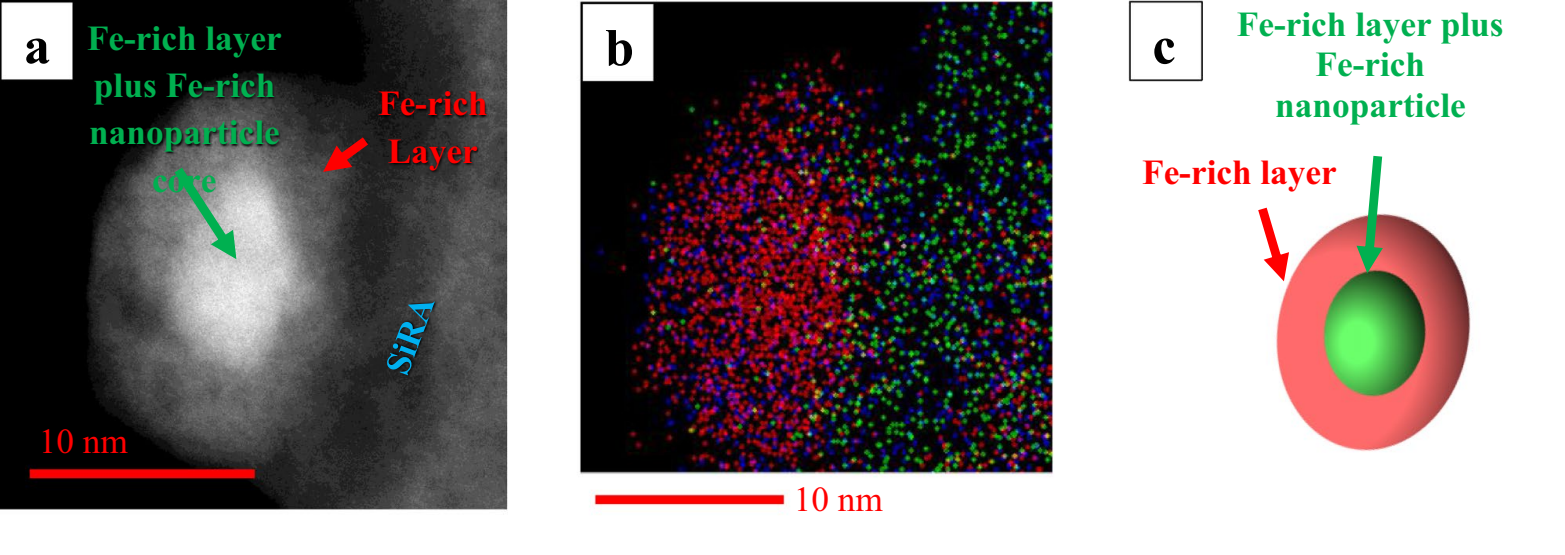
The structure of a nanoparticle lying on the SiRA of an interacted Ath particle extracted from AECs viewed in acSTEM-HAADF mode. (**a**) Core (bright grey) with an average Fe-valence state of 2.04, representing a mixture of core and layer (green arrow), and a “layer” (red arrow) with an average Fe-valence state of 2.40; (**b**) Fast EDXS map of the area seen in a) showing the Fe (red), O (blue), and Si (green) signals; (**c**) Simplified structure of a nanoparticle.

To investigate possible differences in Fe-valence state and stoichiometry between the Fe-rich objects at the modified surface of the extracted amphiboles, we combined the ac-STEM-EDX and the Dual-EELS results (with an estimated overall lateral spatial resolution in the nanometer range, taking into account beam-broadening and possible electron-channeling effects). This complex analysis was performed on a region near the surface of an Ath particle, where it was possible to find a Fe-rich nanoparticle and layer suitable for applying all the described techniques. Figure 6a shows such an Fe-rich nanoparticle and the surrounding amorphous Fe-rich layer. The Fe-valence state at the core of the Fe-rich nanoparticle (green arrow, Fig. 6) is 2.04 ± 0.04, whereas that in the surrounding Fe-rich layer (red arrow) is 2.40 ± 0.09. The valence state of the Fe-rich nanoparticle was evaluated by considering the thickness ratio of the Fe-rich layer to the enclosed nanoparticle. Characterization by acSTEM-EDXS revealed a chemical composition of the Fe-rich layer (Fe = 64.33 ± 1.29 wt%; O = 35.67 ± 0.71 wt%) that is stoichiometrically compatible with ferrihydrite ((Fe^3+^_10_O_14_(OH)_2_)), whereas the chemical composition of the Fe-rich nanoparticles (Fe 83.78 ± 1.68 wt%; O = 16.22 ± 0.32 wt%) is stoichiometrically compatible with wüstite (FeO). Other O-bonding elements were not detected even using Dual-EELS (Supplementary Information Fig. S.3.4), but they might be present in traces. It must be noted, however, that, although stoichiometrically compatible with the compositions of these minerals, both the Fe-rich nanoparticles and the Fe-rich layer are amorphous, as documented by acSTEM observations (e.g., Fig. 6a). The matrix with which the nanoparticles and the Fe-rich layer are in contact is probably a mixture of SiRA and possibly Fe^3+^-silicates, as evidenced by EDX spectra (Supplementary Information Fig. S.3.3, Fig. S.3.5). Of note is the absence of Fe-phosphates or other phosphates in the modified amphibole particle population and, specifically, in the Fe-rich layer (SupplementaryInformation Fig. S.3.3, Fig. S.3.4). This result allows us to infer that the observed particle transformations probably took place within the acidic compartments of the AECs (e.g. phagocytic compartments, lysosomes) and/or that the physicochemical conditions did not allow for phosphorus adsorption or surface coordination. Furthermore, the absence of phosphorus highlights an important difference between the internalized amphiboles and the extracellular ABs described in the literature cited above.

## Discussion

Our study has documented that amphibole particles exhibit great bulk stability down to the near-atomic- and nano-scale within AECs, at least up to 48 h of interaction. Our results, however, also demonstrate that during the interaction with the AECs, the amphiboles were modified at their surface, thus confirming the validity of a previous model of amphibole alteration, which was based on experiments in simulated body fluids^38–41^. Our study, on the contrary, is based on cell-culture exposure experiments, which allows us to propose an expanded conceptual, albeit speculative model by including details observed at the near-atomic- to the nano-scale on AEC-internalized amphibole particles. Based on our data and the related literature^38–41^, our expanded model of amphibole alteration within AECs can be described as follows:

(a) In nature, crystalline amphibole particles are in some cases either fully or partially covered by a heterogeneous SiRA layer, with or without Fe-oxyhydroxide species near its surface^31–33^. Fe-rich amorphous nanoparticles have never been reported at the grain boundary of natural (i.e., environmentally weathered) amphiboles (in our case, the starting material) within the Fe-rich layer (when present).

(b) Once taken up by AECs, the amphibole particles undergo incongruent dissolution, which is analogous to what was observed abiotically by Pacella et al.^39,41^, and Andreozzi et al.^40^ in both an acidic and a neutral pH medium. This process depends, amongst other parameters, on the chemical composition of the amphibole and on the presence or absence of an amorphous, altered surface layer. In our case, in contrast to what has been observed abiotically^38–41^, Mg^2+^ leaching seems to have been more intense for Ath than for Gru and Amo (see Table 2).

(c) During the incongruent dissolution, the grain boundary becomes progressively enriched in Fe. This process causes the exposure at the particle boundary of Fe^2+^-containing structural sites and the erosion of the Fe^3+^-rich outer layer (if present)^41^, and is associated with a lowering of the average valence state of the surface layer (although the difference is mostly not statistically significant when observed on areas of 25 × 25 nm by Dual-EELS, see Table 3). In this first stage, the boundary dissolution is faster than the boundary oxidation. In agreement with Pacella et al.^38,39^, Andreozzi et al.^40^, and Vigliaturo et al.^42^, we do not expect Fe to be released into solution (or only in extremely small amounts), unless the particles are internalized in acidic compartments at a certain point during the experiment (see also Pacella et al.^41^). In that case, the finite-volume compartment (e.g., a lysosome) containing the particle would be easily Fe-saturated, promoting re-precipitation of Fe. The amphibole dissolution and release of Fe in an acidic medium would be consistent with the abiotic experiment conducted by Pacella et al.^41^. The boundary Fe^2+^ is oxidized to Fe^3+^ within or below the altered amorphous surface layer to form Fe^3+^-silicates and, subsequently, Fe^3+^ oxyhydroxides and/or Fe phosphates^38–41^. At this stage, the boundary oxidation has become faster than the boundary dissolution, possibly also as a consequence of the Fe-saturation in the lysosomes. The notable absence of Fe phosphates in our samples, however, is the most important chemical difference between our cell experiments and those performed in simulated biofluids^38–41^, and is probably due to the segregation of the amphibole particles into acidic cell compartments (i.e., lysosomes); alternatively, it could be due the absence of the appropriate physicochemical conditions to allow for phosphorus adsorption or surface coordination.

(d) Simultaneously with the oxidation of the Fe^2+^ at the particle boundary, the amphibole particles dissolve, thereby partially or completely removing the outermost altered SiRA layer containing Mg^2+^, Ca^2+^, and Na^+^, and dissolving the silicate structure^38–40^. This process results in an amphibole grain boundary region, in which the crystalline bulk may be only partially covered by an amorphous layer (see, e.g., Fig. 2d) or nearly devoid of such a layer (see, e.g., Fig. 2a,b).

(e) Formation of Fe^2+^-oxide clusters within the outermost Fe^3+^-rich layer (Fig. 4a).

(f) Growth of Fe^2+^-oxide clusters into amorphous Fe^2+^-oxide nanoparticles, which are embedded in the Fe^3+^-rich layer (mostly containing Fe^3+^-rich oxyhydroxides) covering the altered SiRA (Fig. 4b).

(g) Partial loss of the Fe-rich layer, resulting in a thinner, residual Fe-rich layer on top of the SiRA, which still surrounds Fe^2+^-oxide amorphous nanoparticles (Fig. 6a).

Here, we highlighted a fundamental difference between the previously studied abiotic systems^38–41^ and our observations in biotic systems (i.e., AECs). In abiotic systems, even at acidic pH^41^, Fe-rich amphibole particles are dissolving faster than Fe-poor amphibole particles. In our study, the amphibole particle alterations were more intense for Ath (lower Fe-content—Table 2), than for Gru, and Amo (higher Fe-content—Table 2). This result can be explained by two separate non-exclusive hypotheses:

(1) In our 48-h experiment, we cannot know at what time a given particle, which we studied after its extraction from the AECs, was internalized by the cell, and at what time point the cell might have died without responding with intracellular changes^43^. Therefore, we do not know exactly how much time was needed for this particle to reach the observed end-state (due to the experimental setup, the maximum time was 48 h), nor is it possible to know whether or not the cells were active during the entire experiment (Preliminary data on cell viability are available in the Supplementary Information S.4 and a dedicated paper in preparation); and.

(2) since a certain end-state of the amphibole surface appears to be able to modulate the reactivity and toxicity of a particle, the cell might be able to recognize the surface state of a given particle before, during or after uptake and internalize the particles following different pathways. Specifically, amphibole particles with a surface that is Fe-poor and/or with highly coordinated Fe atoms (e.g., our Ath) could be released into the cytoplasm after internalization by endocytosis (possible phagocytosis) and/or engulfment^44–46^ so that the Fe-undersaturated environment can promote the alteration of the particle boundary to a poorly reactive or unreactive state (i.e.,^33^). In contrast, amphiboles with an Fe-rich surface and/or with poorly coordinated Fe atoms could be segregated into a vesicle that provides a nearly Fe-saturated environment so that they can be stabilized by forming an Fe-rich layer that fully covers the particle (as described in detail by Fantauzzi et al.^33^) within an isolated compartment without affecting the surrounding DNA material and organelles.

If our second hypothesis is further confirmed, this could be solid proof that the “vital effect”^47^ plays a major role in determining the overall reactivity and toxicity of internalized exogenous minerals, whereby the cell is selecting the environment into which a certain particle will be segregated (i.e., Fe-unsaturated cytoplasm versus nearly Fe-saturated vesicle). It could also emphasize that the vital effect’s action is dependent on the initial surface features of the internalized micro- or nano-particles, which can be “recognized” early by the target cells, thus determining the chemical environment that drives the internalized particle transformations (through BIM) as well as the stabilization to a certain physicochemical end-state.

The amphibole particles characterized in this study after their extraction from the interior of the AECs are distinct from the well-known ABs^48,49^, which take longer (i.e., 4 weeks or more) to form in vivo around asbestos^30,50–54^, and are mostly visible in thin sections of lung tissue where they can be studied by optical microscopy, SEM and, less frequently, by TEM^29^. Our research highlights several notable differences between ABs and the altered amphibole particles extracted from AECs studied here:

1. Location where the Fe-rich layer of the amphibole particles is produced: in our study, we focused on the amphibole particles that were internalized and transformed *within* the AECs, whereas ABs are commonly generated in contact with the tissue of the lower respiratory tract^55,56^ after phagocytosis and failure of lung clearance^57–64^. The fact that our high-resolution methods did not detect P within the Fe-rich objects studied here (i.e., layer, clusters, and nanoparticles) suggests that the particles were internalized within acidic compartments (e.g., lysosome), at least for a certain amount of time.
2. Formation mechanism: ABs are formed when the lung-clearance mechanism fails, resulting in “asbestos” particles being coated with an Fe-protein-mucopolysaccharide^57–64^. This coating can be generated through BCM over a period of 4 weeks or more. These formation pathways of ABs are, thus, different from the amphibole-alteration mechanism described here, which took place within AECs and during a much shorter interaction time, which points to abiotic or, possibly, BIM processes.
3. Chemical composition: we have shown that the Fe^3+^-rich amorphous layer, as well as the Fe-rich nanoparticles and clusters in this surface layer, consist primarily of Fe (with O and H), with no detectable P. Asbestos bodies are also rich in Fe, but additionally contain substantial amounts of P, Ca, and Mg^65–67^, whereby Ca acts as an aggregation agent for ferritin^64^ and P is associated with the ferritin core^65^. Moreover, Fe is not a dominant component of the ABs formed around crocidolite (i.e., asbestiform riebeckite amphibole), chrysotile (a serpentine mineral), and/or erionite (a zeolite) ^68^.
4. Origin of Fe in the layer that forms around amphibole particles: in our study, the Fe-rich layer with its enclosed clusters and nanoparticles is most likely derived from the underlying amphibole itself. The most chemically modified amphibole (EDXS bulk analysis, and surface modification imaging), Ath, is the one that presents more abundant Fe-rich nanoparticles within a thicker modified Fe-rich layer compared to the other two amphiboles. In the case of ABs, Fe is mostly derived from the Fe-protein-mucopolysaccharides^57–64^.

## Methods

### Studied samples

The three starting materials for our experiments are the UICC (Union for International Cancer Control) amosite (Amo), described in detail by Pollastri et al.^69^ and Vigliaturo et al.^32^, and two non-asbestiform amphiboles: anthophyllite (Ath) from Kongsberg (Norway), labelled MNHN 29_102, and grunerite (GRU) from Salem (India), labelled MNHN 97_373, both part of the mineralogical collection of the Muséum National d'Histoire Naturelle in Paris (France). Ath and Gru were fully characterized in this study (Supplementary Information S.1) by using X-Ray Powder Diffraction (XRPD), Fourier-Transform Infrared (FTIR) and Raman spectroscopies, Mössbauer Spectroscopy and Electron Probe Micro-Analysis (EPMA). As presented elsewhere, Ath and Gru were found to consist mainly of bladed and acicular crystallites, whereas the UICC standard Amo was mainly asbestiform, but also showed some cleavage fragments (Vigliaturo et al., in prep.).

### Cell treatment and sample preparation for acS/TEM

The three different amphiboles were sterilized in 1.5 mL Safe Lock tubes (Eppendorf, Germany) for 20 min at 121 °C (VX-150, Systec, Germany) before each use with cells to ensure their sterility. The minerals were then suspended in A549 cell growth medium up to a desired maximum concentration (100 µg/mL) with two 5-min sonic bath cycles interspaced with a 2-min vortexing step.

The AECs were seeded on 12-well plates at a density of 6.6 × 10^5^ cells per well one day prior to any experiment. Cells were treated with each of the three amphiboles at a final concentration of 50 µg/mL and incubated for 48 h at standard culture conditions. After incubation, the medium was changed with a fresh one without mineral particles, so that non-internalized extra-cellular amphibole particles were washed away and not studied. Subsequently, the cells were detached using a cell scraper.

The mineral particles were extracted from the AECs with a chemical digestion procedure using a gentle bleach method^63,70,71^: the cells were transferred into a 50 mL tube containing 10 mL of NaClO (14 vol.%), then vortexed and left to rest for 30 min. The suspension in the tube was then centrifuged at 80,000 RPM, and the NaClO substituted by deionized water. Subsequently, the material was transferred onto lacey-carbon copper grids (SPI Supplies) for acS/TEM (aberration-corrected Scanning Transmission Electron Microscopy) investigations.

### Nanoscale investigations: acS/TEM-EDXS and Dual-EELS

The nanoscale investigations were conducted using an atomic-resolution acSTEM (ARM 200 F, JEOL), equipped with a high-brightness Cold Field Emission Gun (CFEG) operating at 80 kV. The microscope was equipped with an EDXS system (Centurio 100 mm^2^, JEOL), and an energy filter (QuantumGIF, Gatan, USA) for Dual-EELS. AcSTEM/TEM-EDXS and Dual-EELS (Electron-Energy-Loss-Spectroscopy) were used systematically to characterize both the natural starting materials and the minerals extracted from the AECs after interaction with cell cultures for 48 h, as summarized in Table 1.

AcSTEM-EDXS spectra were collected to compare the chemical composition of the starting materials with that of the particles after interaction with the AECs. We recorded maps to obtain the chemical composition of at least 100 particles (dwell time 2 ms per pixel) for each sample. The reduced electron density (Spot size 6) and voltage (80 kV) allowed us to minimize beam damage, dispersion effects, and the evaporation of volatile elements^32,72^.

### Artefact tests

The physicochemical state of the surface depends on the conditions to which an amphibole particle was exposed either in the environment or during sample preparation and investigation with an electron beam. Because artefacts at this level of investigation may be generated, all our materials were tested for electron beam stability and for the possibility of modification by the surrounding medium (Supplementary Information S.5).

## Supplementary Information

Supplementary Information.

## Data Availability

All data generated or analyzed during this study are included in this published article (and its Supplementary Information files).
