# Supplementary material for: Nanoscale transformations of amphiboles within human alveolar epithelial cells
Source: Sci Rep. 2022 Feb 2;12:1782. doi: 10.1038/s41598-022-05802-x (PMC8810849; doi:10.1038/s41598-022-05802-x)
Supplement: Supplementary file 1 — Supplementary Information. [file 41598_2022_5802_MOESM1_ESM.pdf]

Supplementary Information for:

Nanoscale Transformations of Amphiboles Within Human Alveolar Epithelial Cells

*Ruggero Vigliaturo[a], Maja Jamnik[b], Goran Dražić[c], Marjetka Podobnik[b], Magda Tušek Žnidarič[d], Giancarlo Della Ventura[e,f,g], Günther J. Redhammer[h], Nada Žnidaršič[i], Simon Caserman[b], Reto Gieré[a,j]

[a] Department of Earth and Environmental Science, University of Pennsylvania, Philadelphia, U.S.A.

[b]Department of Molecular Biology and Nanobiotechnology, National Institute of Chemistry, Ljubljana, Slovenia

[c]Department of Materials Chemistry, National Institute of Chemistry, Ljubljana, Slovenia

[d]Department of Biotechnology and System Biology, National Institute of Biology, Ljubljana, Slovenia

[e]Department of Geological Sciences, University of Roma Tre, Roma, Italy

[f]INFN-Istituto Nazionale di Fisica Nucleare, Frascati (Rome), Italy

[g]INGV, Via di Vigna Murata 605, 00143 Roma, Italy

[h]Department of Materials Science & Physics, University of Salzburg, 5020 Salzburg, Austria

[i]Department of Biology, Biotechnical Faculty, University of Ljubljana, Ljubljana, Slovenia

[j]Center of Excellence in Environmental Toxicology, University of Pennsylvania, U.S.A.

*Corresponding author: Ruggero Vigliaturo, ruggero.vigliaturo@gmail.com

S.1 Starting material: mineralogical and crystal-chemical characterization

Supplementary methods S.M.1.1 XRPD

The XRPD data were collected using a Scintag X1 diffractometer (ThermoFisher, Waltham, MA, USA) equipped with Cu-Kα_1 radiation source (λ = 1.54055 Å, 40 mA, 45 kV), fixed divergence slits, and a Peltier-cooled Si (Li) detector with a resolution of < 200 eV. A divergent slit width of 2 mm and a scatter-slit width of 4 mm were used for the incoming beam, whereas a receiving slit-width of 0.5 mm and scatter-slit width of 0.2 mm were used for the diffracted beam. The data were acquired in a step-scan mode in the 2–70° 2θ range, with a step size of 0.05° 2θ, and a counting time of 3 s/step.

Supplementary methods S.M.1.2 FTIR Spectroscopy

The FTIR spectra of amphibole powders were collected using a Nicolet iS50 spectrometer, equipped with a Globar source, a KBr beam-splitter and a DGTS (Deuterated Triglycine Sulphate) detector. The powdered samples were prepared as KBr pellets, using a ratio of 5:150 and 0.5:150 mg for the regions 3000–4000 cm^{-1} and <1200 cm^{-1}, respectively.

Single-crystal FTIR spectra in the OH-stretching medium-infrared (MIR) range were collected with unpolarized light using a Bruker Hyperion 3000 microscope equipped with a MCT (Mercury Cadmium Telluride) detector and a KBr beam-splitter.

Supplementary methods S.M.1.3 Raman spectroscopy

Unpolarized Raman spectra were excited with a 532 nm laser, an integration time of 5 s per scan, averaging 5 scans per point, and with a 5x objective; the laser power was set to 20 mW. The wavenumber accuracy was ±0.5 cm^{-1}, and the spectral resolution was 1 cm^{-1}.

Supplementary methods S.M.1.4 Mössbauer Spectroscopy

^{57}Fe Mössbauer spectra were acquired at room temperature with an instrument in horizontal arrangement (^{57}Fe Co/Rh single-line thin source, constant acceleration mode with symmetric triangular velocity shape, multi-channel analyzer with 1024 channels, and regular velocity calibration against metallic Fe). Data evaluation was performed using the RECOIL program suite[73, 74]. All spectra were corrected for thickness effects and then analyzed using the full static hyperfine interaction Hamiltonian analysis with Lorentzian-shaped doublets.

Supplementary methods S.M.1.5 EPMA

EPMA data for the studied amphiboles were collected by wavelength-dispersive X-ray spectroscopy techniques using an electron microprobe CAMECA SX50 (CAMECA, Gennevilliers, France). Analytical conditions were: 15 keV accelerating voltage, 20 nA beam current, 5 µm beam size, and 100 s counting time. The data reduction was made using the ZAF4/FLS software (standard version) by Link Analytical (Oxford, UK). Compositions were determined relative to the following natural and synthetic standards: diopside (Si, Mg, Ca), metal oxide (Ti, Fe, Mn, Cr), orthoclase (Al, K), albite (Na), and fluorite (F).

S.1.1 Anthophyllite – XRPD

The XRPD data indexed for comparison with PDF card #451343 are compatible with orthorhombic *Pnma* symmetry.

S.1.2 Anthophyllite – FTIR

The OH-stretching FTIR spectrum shows two main bands due to the distribution of Mg and Fe^{2+} at the *M*(1,3) sites; each of these bands is split into two hyperfine components due to the presence of two crystallographically independent OH groups in the orthorhombic structure (Fig. S.1.2a). The most intense doublet at 3670-3665 cm^{-1} is assigned to the local MgMgMg-OH configuration around the O–H group, whereas the lower-intensity doublet at 3655-3650 cm^{-1} is assigned to the local MgMgFe–OH configuration around the O–H dipole. The relative integrated intensities (areas below the curves) of the doublets, calculated by neglecting the hyperfine splitting (Fig. S.1.2b) according to the method explained in Della Ventura[75], yields an Fe^{2+} content at *M*(1,3) = 0.21 apfu (atoms per formula unit).

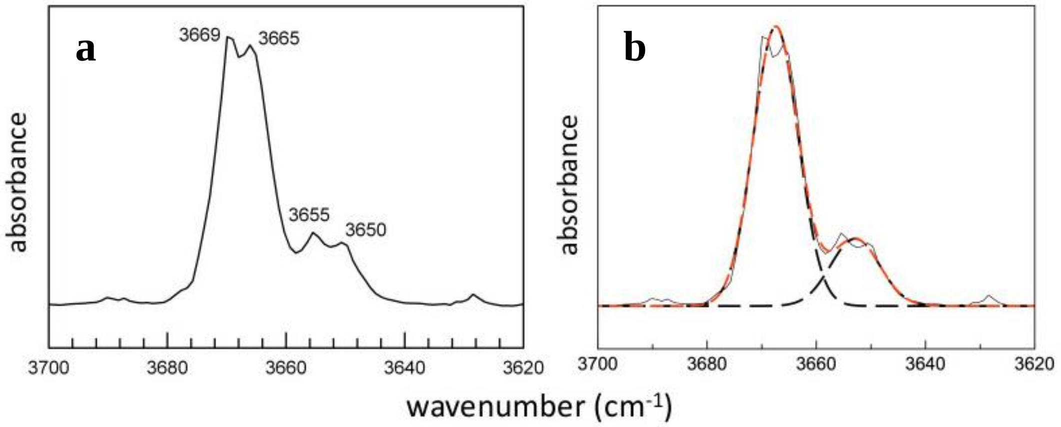

Fig. S.1.2 FTIR spectrum of anthophyllite MNHN 29_102. **a)** the OH-stretching spectrum; **b)** the spectrum decomposed using two Gaussian components and neglecting the hyperfine splitting.

S.1.3 Anthophyllite – Raman spectroscopy

The Raman spectra (Fig. S.1.3) have been collected in both the low-frequency (a) and the OH-stretching (b) regions. The latter is identical to the IR spectrum; the band splitting is also partially resolved. From the integrated intensities we derived Fe^{2+} at *M*(1,3) = 0.22 apfu, i.e., the same value as the one determined via IR.

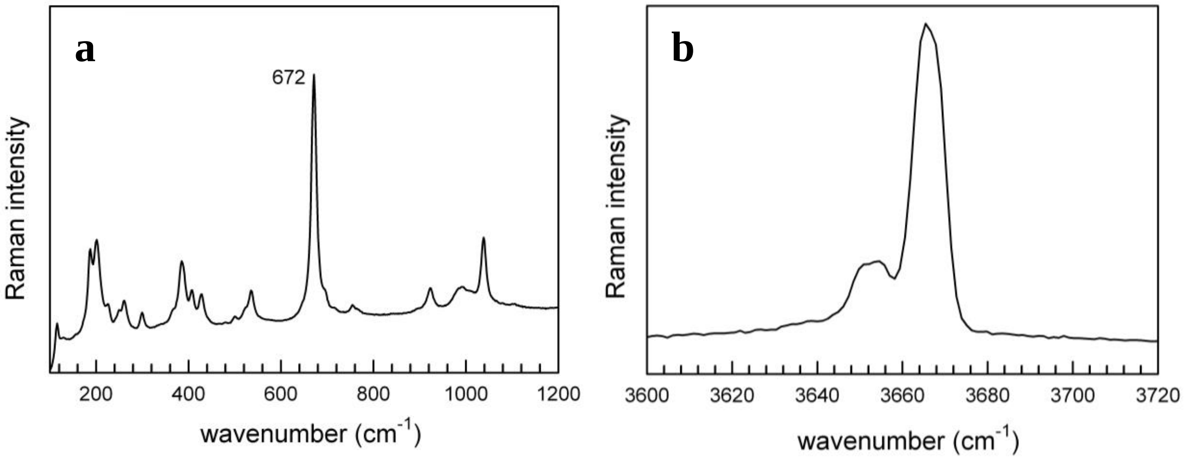

Fig. S.1.3 Raman spectrum of anthophyllite MNHN 29_102. **a)** low-frequency region; **b)** OH-stretching region.

The Raman spectrum collected in the lattice-mode region (< 1200 cm^{-1}) is virtually identical to the patterns published in the literature for both fibrous[76] and non-asbestiform[77] anthophyllite, stressing the validity of Raman spectroscopy as a tool for identifying amphibole species. In particular, the "ring-breathing mode" is observed at 672 cm^{-1}, a frequency value characteristic for anthophyllite[76].

S.1.4 Anthophyllite – Mössbauer spectroscopy

The ^{57}Fe Mössbauer spectrum (Fig. S.1.4) can be evaluated using three doublets corresponding to Fe^{2+}. The dominant Fe^{2+} doublet with an isomer shift of 1.123(9) mm/s and a small quadrupole splitting of 1.840(2) mm/s is assigned to the *M*(4) site, based on literature data for similar compounds[78, 79, 80, 81]. The two remaining Fe^{2+} doublets correspond to Fe^{2+} at the *M*(1) and the *M*(2, 3) sites, respectively and show low relative absorption. The refinement of only two Fe^{2+} doublets leads to an unsatisfactory fit of the high-energy flank of the absorption line, which is centred at +2.0 mm/s. Using the three-Fe^{2+}-doublet model, some small residuals are present at velocities around +0.7 mm/s, which indicate very low Fe^{3+} concentrations. Adding a 4th doublet yields typical hyperfine parameters for Fe^{3+} with a relative area fraction of ~ 1.4 % (Table S.1.4).

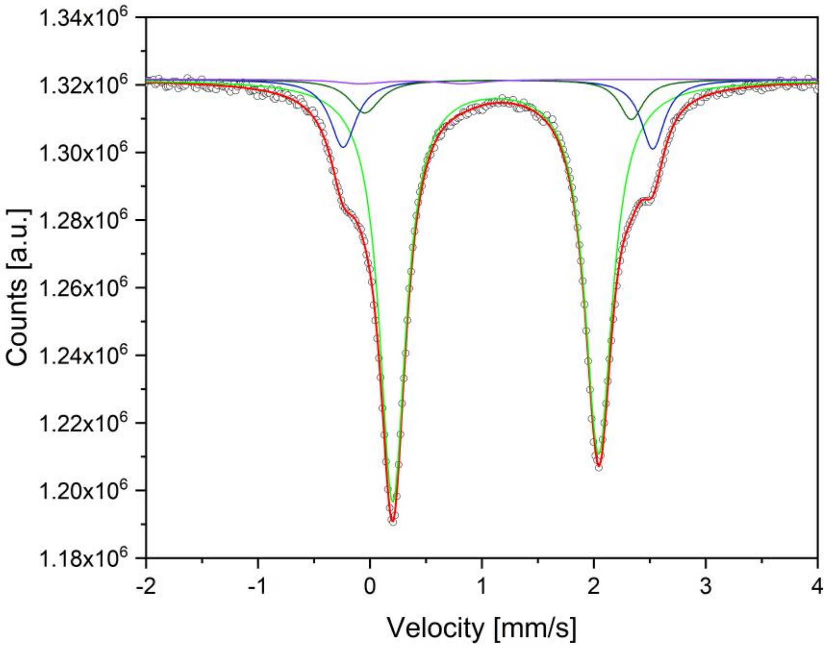

Fig. S.1.4 The fitted Mössbauer spectrum of anthophyllite MNHN 29_102

Tab. S.1.4 Results of analysis of the Mössbauer spectrum of anthophyllite MNHN 29_102. IS = isomer shift (mm/s), QS = quadrupole splitting (mm/s), HWHM = Half width at half maximum

**Assignment**	**IS δ (mm/s)**	**QS Δ (mm/s)**	**HWHM (mm/s)**	**Relative Area (%)**	**Fe (apfu)**
Fe^{2+} *M*(4)	1.123(9)	1.840(2)	0.149(16)	79.6(9)	1.19
Fe^{2+} *M*(1)	1.144(5)	2.766(4)	0.127(12)	12.6(14)	0.19
Fe^{2+} *M*(2,3)	1.144(4)	2.381(5)	0.129(12)	6.4(12)	0.10
Fe^{3+}	0.375(9)	0.92(9)	0.244(16)	1.4(6)	0.02

Taking into account a total FeO content of 13 wt% (see EPMA data below – Section S.1.9), corresponding to 1.50 apfu based on 23 oxygen atoms, we can calculate the iron species from the relative areas of the Mössbauer doublets, and distribute them among the available structural sites; the results are listed in Table S.1.4. The data show that the Fe^{3+} content is negligible (0.02 apfu); most ferrous iron is ordered at the *M*(4) site (1.19 apfu), whereas a small fraction (0.29 apfu) is disordered at *M*(1,2,3) sites. Due to its specific cation distribution, in particular the strong ordering of Fe^{2+} at the *M*(4) site, this amphibole probably necessitates establishing a new root-name[82]. Accordingly, a proposal for this nomenclature modification is under evaluation of the IMA (International Mineralogical Association) Subcommittee on Amphibole Classification.

S.1.5 Grunerite – XRPD

The XRPD data, indexed for comparison with PDF card #441401, are compatible with monoclinic *C*2*/m* symmetry.

S.1.6 Grunerite – FTIR

The OH-stretching FTIR powder spectrum (Fig. S.1.6) of grunerite MNHN 93_373 shows four bands at 3666, 3652, 3636 and 3618 cm^{-1}, due to the distribution of Mg and Fe^{2+} at the *M*(1,3) sites. Based on the vast literature summarized in Hawthorne & Della Ventura[83], these peaks are assigned to the different cationic configurations around the O–H group resulting from the distribution of two atoms over three structural sites. From the relative integrated intensities of the four peaks, the amount of Mg and Fe^{2+} at *M*(1,3) can be determined. The spectrum in Figure S.1.6a was modelled using four Gaussian components[12 – main text, 75]. For convergence, small residuals on the lower wavenumber side of the main peaks required the addition of smaller components for refinement; this is due to the presence of Fe^{2+} at the *M*(4) site[79]. The relative intensities of the four main components allow for the derivation of an Fe^{2+} content of 2.19 apfu at the *M*(1,3) site.

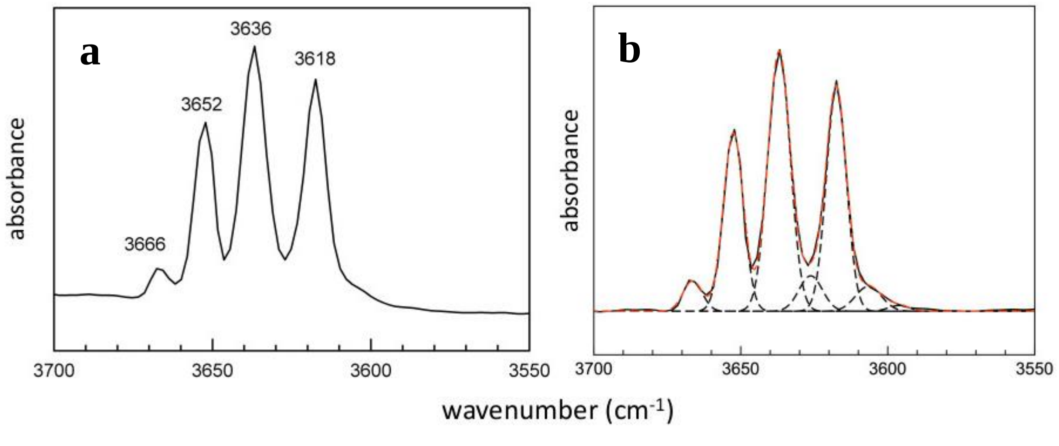

Fig. S.1.6 FTIR spectrum of grunerite MNHN 97_373. **a)** the OH-stretching spectrum; **b)** the spectrum decomposed using Gaussian components.

S.1.7 Grunerite – Raman spectroscopy

The Raman spectrum in the OH-stretching region (Fig. S.1.7b) shows the four-band pattern already described for the FTIR spectrum. The relative intensities of the four components are somehow different from those observed in the IR pattern, due to a strong effect of crystal orientation under the laser beam[77]. The low-frequency region (Fig. S.1.7a) is virtually identical to the spectrum given for amosite in Rinaudo et al.[76], with the characteristic "ring-breathing mode" at 661 cm^{-1}.

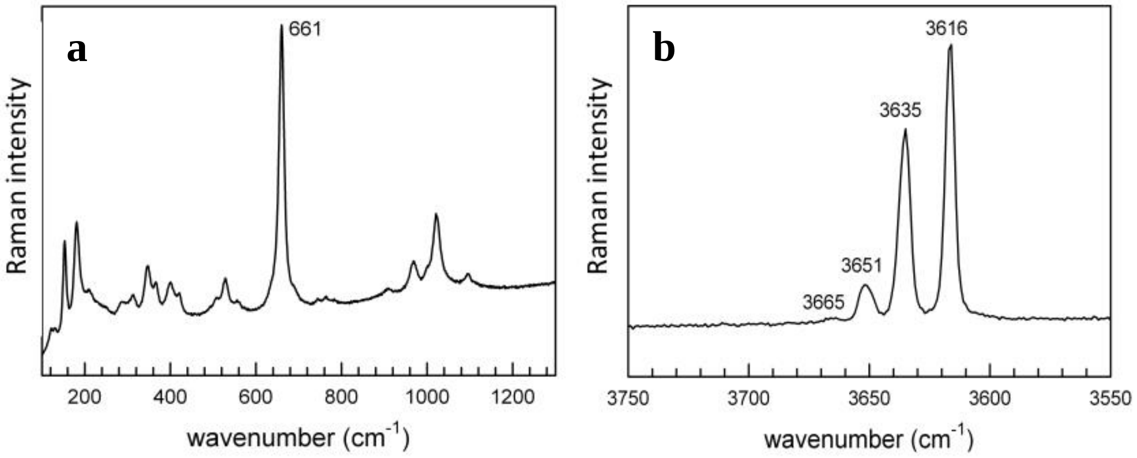

Fig. S.1.7 Raman spectrum of grunerite MNHN 97_373. **a)** low-frequency region; **b)** OH-stretching region.

S.1.8 Grunerite – Mössbauer spectroscopy

The ^{57}Fe Mössbauer spectrum of the grunerite sample is best evaluated with three doublets for Fe^{2+} (Fig. S.1.8); refined parameters are given in Table S.1.8. The inner Fe^{2+} doublet, which is well separated from the other two outer lines, is assigned to the *M(*4) site based on its Mössbauer parameters. The two additional doublets, due to Fe at the *M*(1,2,3) sites, can be assigned on the basis of the work of Linares et al.[84]; accordingly, the doublet with the larger quadrupole splitting (~2.842 mm/s) is assigned to Fe^{2+} at *M*(1,3), whereas the one at 2.683 mm/s is assigned to Fe^{2+} at *M*(2).

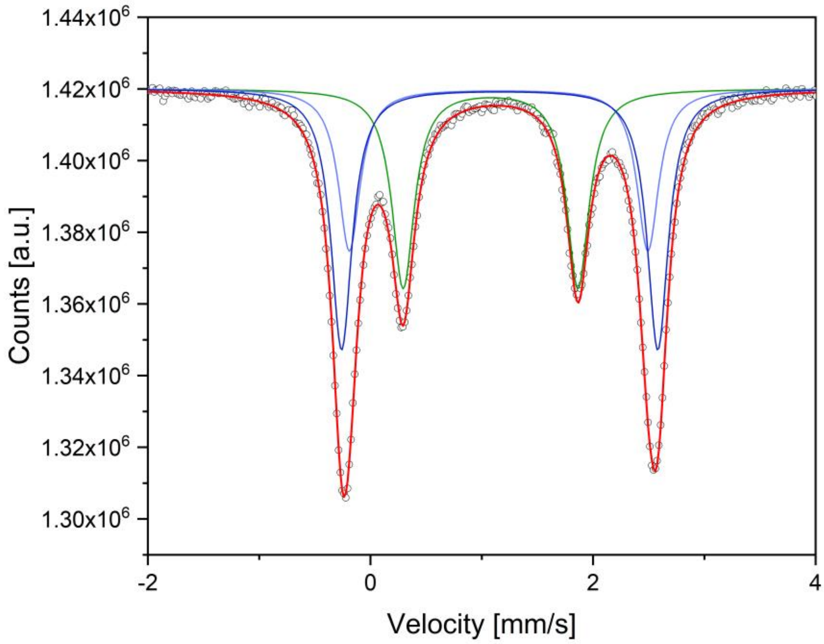

Fig. S.1.8 The fitted Mössbauer spectrum of grunerite MNHN 97_373

Tab. S.1.8 Results for the deconvolution of the Mössbauer spectrum of grunerite MNHN 97_373. IS = isomer shift (mm/s), QS = quadrupole splitting (mm/s), HWHM = Half width at half maximum

**Assignment**	**IS δ (mm/s)**	**QS Δ (mm/s)**	**HWHM (mm/s)**	**Relative Area (%)**	**Fe (apfu)**
Fe^{2+} *M*(2)	1.152(4)	2.683(4)	0.118(3)	26.2(7)	1.38
Fe^{2+} *M*(1,3)	1.162(3)	2.842(2)	0.115(3)	40.9(6)	2.15
Fe^{2+} *M*(4)	1.079(2)	1.571(2)	0.121(2)	32.9(3)	1.73

By considering a total FeO content of 39.42 wt% (see EPMA data below), corresponding to 5.25 apfu on the basis of 23 oxygen atoms, we calculated the Fe distribution reported in Table S.1.8. The data show that most Fe^{2+} is almost equally disordered at the *M*(1,2,3) sites, whereas a significant fraction of the Fe^{2+} (1.73 apfu) is allotted at the *M*(4) site (Table S.1.8). It is worth noting that the Fe^{2+} value at *M*(1,3) provided by fitting the OH-stretching spectrum (see section S.1.6) is in excellent agreement with that resulting from Mössbauer spectroscopy.

S.1.9 EPMA Data

Tab. S.1.9 EPMA data (in wt%) for anthophyllite and grunerite used in our experiments. The EPMA data for the amosite sample are reported in Pollastri et al.[69 – main text]. Note that according to the nomenclature of amphiboles (e.g., [82]) the *C*-cations include all octahedral cations at the *M*(1,2,3) sites, whereas the *B*-cations include all cations at the *M*(4) site.

	Anthophyllite			Grunerite		
	**Mean (*n* = 8)**	**Max**	**Min***	**Mean (*n* = 11)**	**Max**	**Min***
**SiO_2**	55.97	56.50	55.35	50.40	50.58	50.07
**TiO_2**	0.10	0.14	0.04	0.02	0.06	0.00
**Al_2O_3**	3.15	3.60	2.90	0.34	0.38	0.32
**FeO**	13.00	13.39	12.75	39.42	39.95	38.87
**Cr_2O_3**	0.05	0.09	0.03	0.01	0.08	0.00
**MnO**	0.42	0.49	0.33	0.10	0.13	0.06
**MgO**	24.81	25.06	24.41	6.41	6.57	6.25
**CaO**	0.58	0.71	0.50	0.56	0.63	0.46
**Na_2O**	0.25	0.29	0.19	0.06	0.10	0.03
**K_2O**	0.01	0.03	bdl	0.01	0.03	bdl
**F**	0.03	0.09	bdl	0.04	0.14	bdl
**Cl**	0.01	0.02	bdl	0.01	0.02	bdl
**Total**	98.33	100.32	96.47	97.37	98.59	96.06
**O_F_Cl**	0.01	0.04	0.00	0.02	0.06	0.00
**Total**	98.32	100.28	96.47	97.35	98.53	96.06
**Si**	7.694			8.030		
**Al**	0.306			0.000		
**Fe^{3+}**	0.000			0.000		
**Ti**	0.000			0.000		
**Σ T site**	8.000			8.030		
**Al**	0.204			0.064		
**Cr**	0.005			0.001		
**Fe^{3+}**	0.140			0.000		
**Ti**	0.010			0.002		
**Mg**	4.640			1.522		
**Fe^{2+}**	0.000			3.410		
**Mn**	0.000			0.000		
**Ca**	0.000			0.000		
**Σ C cations**	5.000			5.000		
**Mg**	0.445			0.000		
**Fe^{2+}**	1.354			1.842		
**Mn**	0.049			0.013		
**Ca**	0.085			0.096		
**Na**	0.067			0.019		
**Σ B cations**	2.000			1.970		
**Na**	0.000			0.000		
**K**	0.002			0.002		
**Σ A cations**	0.002			0.002		
**Σ cation**	15.002			15.002		
**Cl**	0.002			0.003		
**F**	0.013			0.020		
**Σ oxygens**	23.000			23.000		
**$Mg/(Mg+Fe_{tot})$**	0.773			0.225		

* bdl = below detection limit

S.2 Starting material: Characterization by aberration-corrected Scanning/Transmission Electron Microscopy combined with Energy-Dispersive X-Ray Spectroscopy and Dual-range Electron Energy-Loss Spectroscopy (acS/TEM EDXS Dual EELS)

In addition to the above methods, acS/TEM-EDXS and Dual-EELS were used to study the starting materials.

S.2.1 acSTEM BF-Medium-Angle Annular Dark Field (MAADF), and acHigh-Resolution (HR)TEM – morphology

The morphology of the starting materials is quite variable despite the purity of the samples. As also shown in the dimensional study (S.2.3), the simultaneous presence of fibers and other morphologies is characteristic for all samples. On the investigated scale, the anthophyllite and grunerite samples primarily consist of bladed and acicular crystals. Amphibole fragments are mostly identifiable as lamellar and bladed morphologies (e.g., Fig.S.2.1.1).

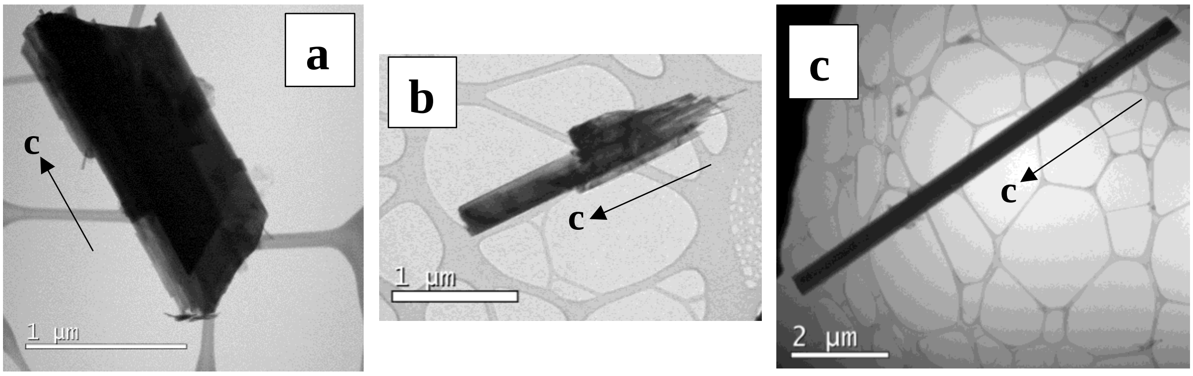

Fig. S.2.1.1 Example of three observed morphologies (TEM): **a)** blade-like anthophyllite particle with stepped lamellar grain boundary; **b)** prismatic-like grunerite particle with a fiber-like apex; and **c)** asbestiform amosite. The arrows labelled with "c" represent the major crystallographic direction of the particle.

The UICC standard amosite particles have the typical asbestiform appearance (Figs. S.2.1.1c, S.2.1.2a), but many fragments accompany the elongated mineral particles (Fig. S.2.1.2a).

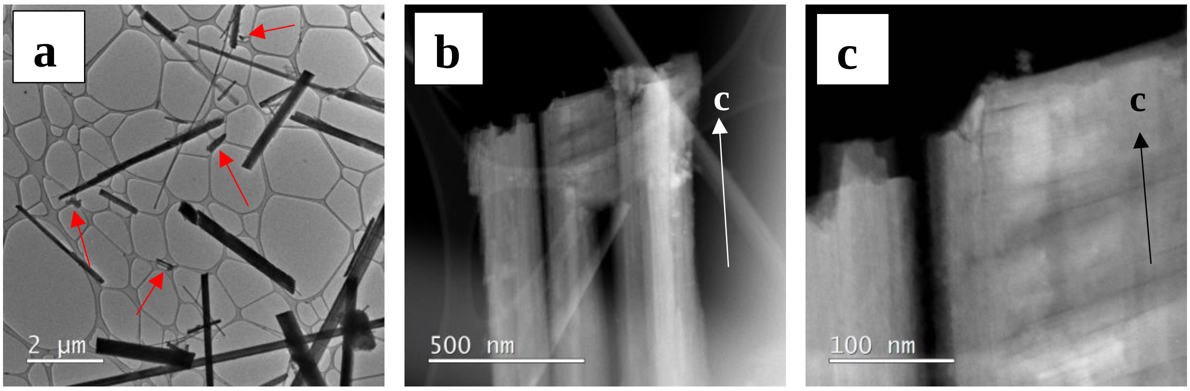

Fig. S.2.1.2 Amosite-asbestos particles showing signs of **a)** cleavage fragments, highlighted with red arrows (TEM); **b)** longitudinal fragmentation (DF-acSTEM); and **c)** randomly oriented cracks (DF-acSTEM). The arrows labelled with “c” represent the major crystallographic direction of the particle.

S.2.2 acHRTEM, SAED, and acSTEM BF-MAADF – crystal structure

The presence of defects can be of pivotal importance in dictating the dissolution pathways of amphiboles, as demonstrated by Crawford[85] for particles that have been exposed to human blood serum[86]. In the starting materials, the cleavage fragments of amosite, as well as the anthophyllite and grunerite particles showed almost no defective structures (as we document in the main text), except for areas with rare lamellar "peelings" (Fig. S.2.2.1) and rare single- and triple-chains (Fig. S.2.2.2).

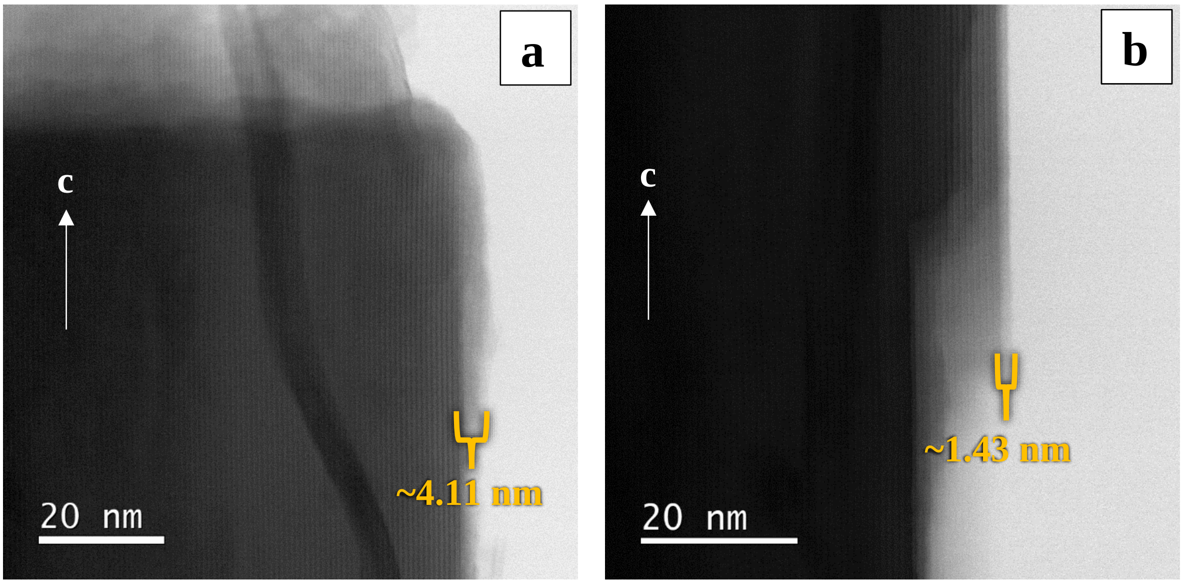

Fig. S.2.2.1 a) acSTEM BF example of an anthophyllite particle showing a defect-free structure and a modified amorphous grain boundary; **b)** acSTEM BF picture displaying a detail of another anthophyllite particle in the same orientation and showing a crystalline grain boundary, which is not covered by amorphous material (i.e. SiRA). The different contrasts are probably related to different thicknesses in this area due to the lamellar stepped morphology of the grain boundaries. The yellow brackets indicate the thickness of the amorphous material. The arrows labelled with "c" represent the major crystallographic direction of the particle.

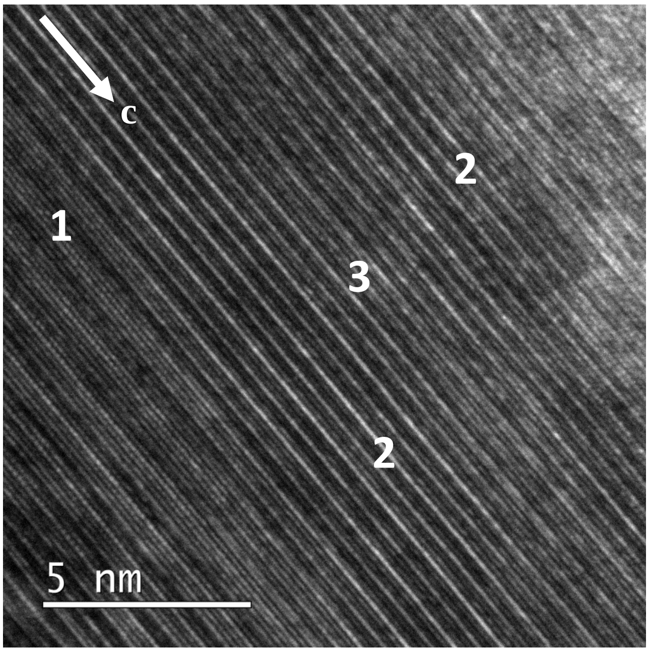

Fig. S.2.2.2 HRTEM picture of a rare defective area where single (1), double (2) and triple (3) chains are visible in a grunerite crystal. The arrow labelled with "c" represents the major crystallographic direction of the particle.

S.2.3 Si-rich amorphous layer (SiRA) – Electron Energy-Loss Spectrum (EELS)

Here we show an example of the EELS spectra collected in a region similar to the one displayed in Fig. 1d of the manuscript. In accordance with the qualitative observation of the spectrum (Fig. 2.3.1), the SiRA contains mostly Si and O, with minor Fe, and possibly traces of K and Ca. The experimental conditions and the type of material (mostly Si and O) does not allow for a reliable quantitative analysis of the elements.

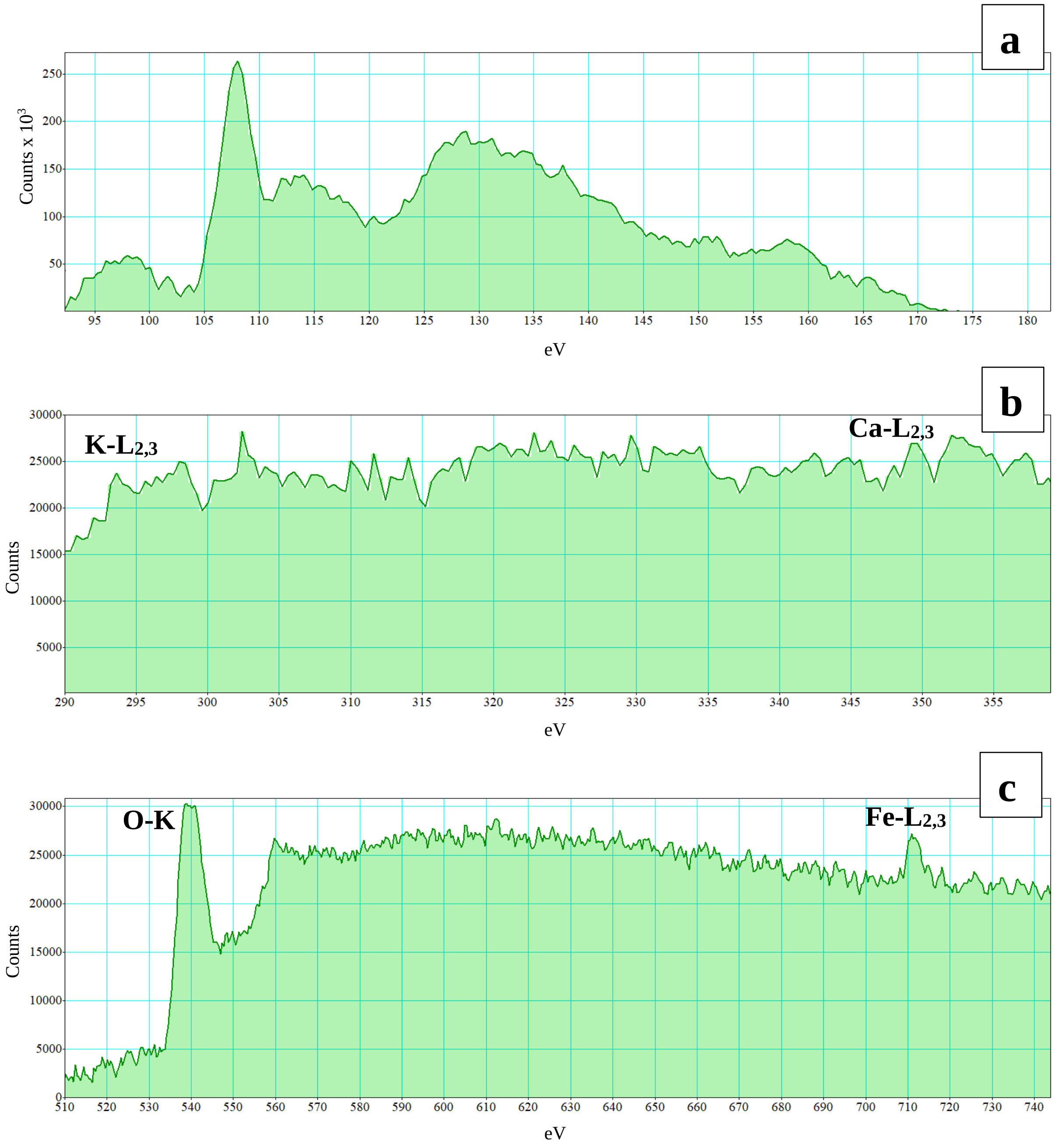

Fig. S.2.3.1 Raw background-subtracted high-loss EELS spectrum collected on the SiRA region **a)** detail of the Si–$L_{2,3}$ edge; **b)** detail of the K– and Ca–$L_{2,3}$ edges (with the expected edge positions labelled) showing noise only and no distinguishable peak; **c)** detail of the O–K and Fe–$L_{2,3}$ edges.

S.3 Transformation of the grain boundaries of amphiboles particles during the interaction with AECs

Various morphologies, habits, and degrees of transformation were detected in particles retrieved from the alveolar epithelial cells.

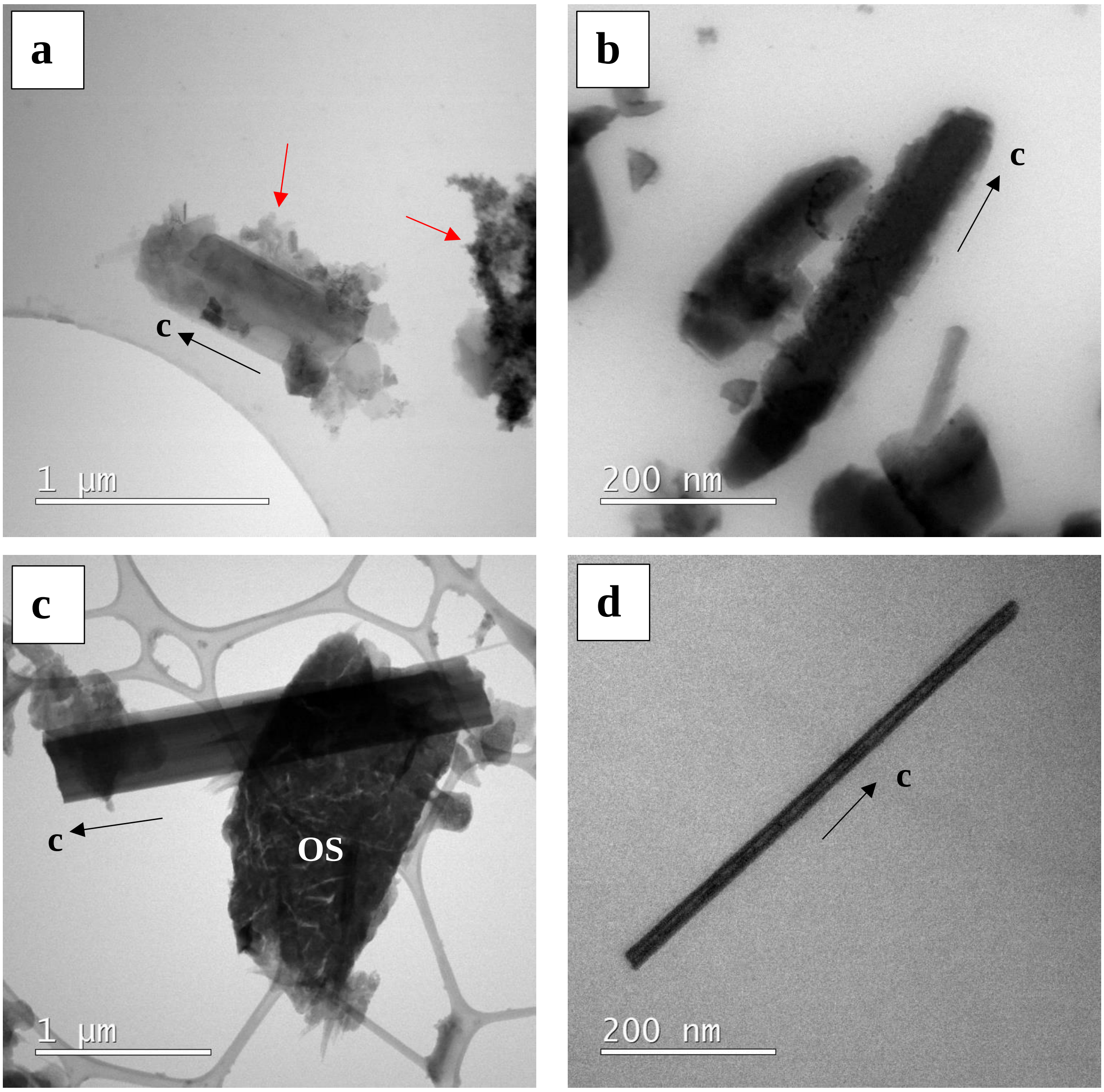

Fig. S.3.1 a) BF image of a blade-like anthophyllite particle (type-2), in which organic material is visible (red arrows); **b)** BF image of an elongated particle of grunerite (type-2) after interaction with AECs displaying rough, highly modified grain boundaries, compromised morphology, and clear signs of dissolution of the amorphous layer (this image is the low-resolution analogue image of Fig. 2d in the main text; **c)** BF image of an elongated particle of grunerite (type-1), which partially overlaps with organic material and salts (labelled as "OS"); d) BF image of an extremely small elongated amosite particle (type-1). The arrows labelled with "c" represent the major crystallographic direction of the particle.

In contrast to the particle core (see main text Fig. 3 and section S.2.2), the particle boundaries can undergo several transformations (Fig. S.3.2).

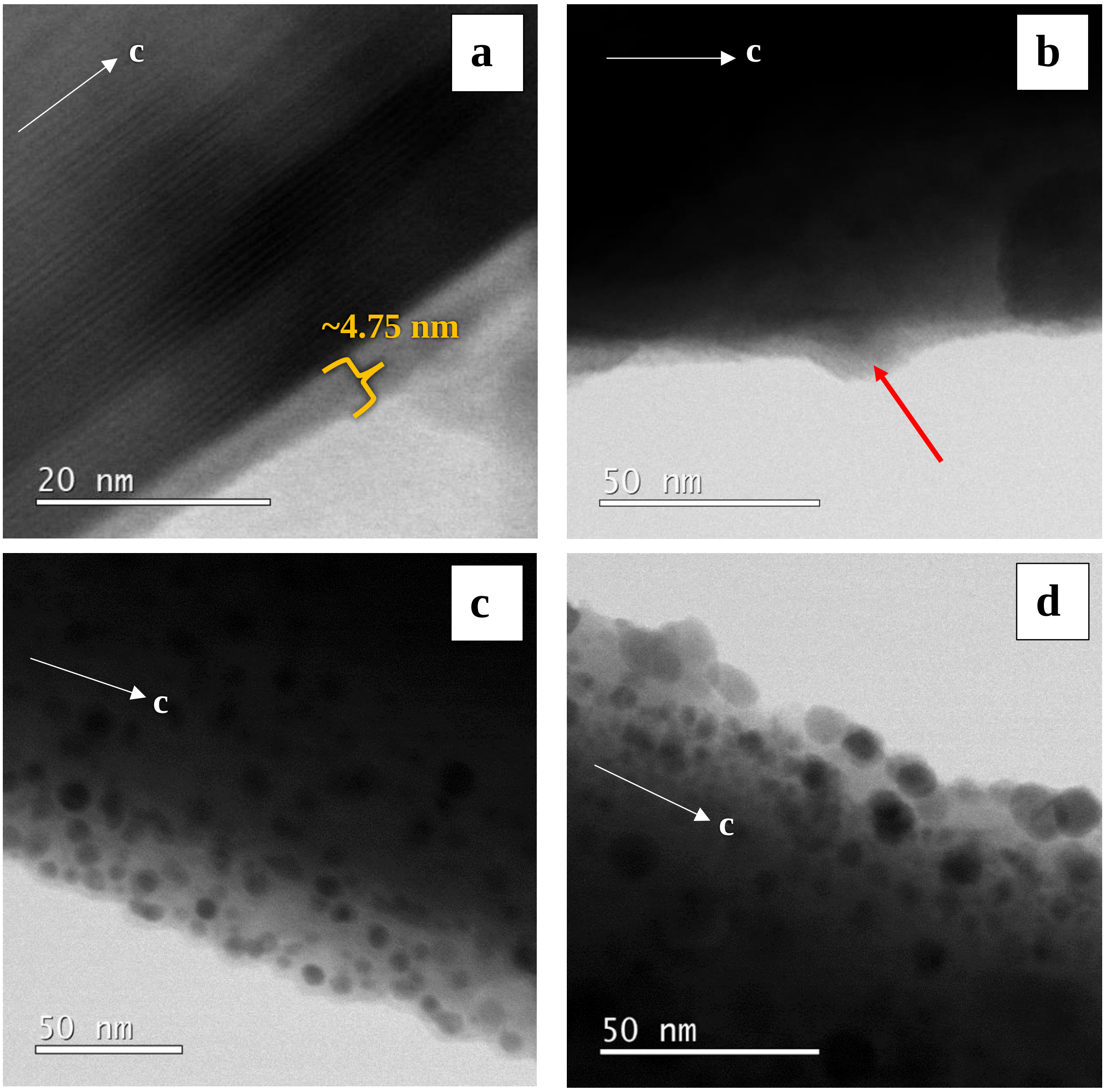

Fig. S.3.2 a) a Gru particle (type-1) showing an amorphous layer at its boundary **b)** an Amo particle (type-1) showing a heterogeneous accumulation of amorphous material at the boundary (red arrow) **c)** and **d)** Low-magnification BF-TEM micrographs of the external modified shell, which contains Fe-rich nanoparticles of different dimensions and with an ellipsoidal shape located at the boundary of Ath particles (type-2). The yellow brackets indicate the amorphous material thickness. The arrows labelled with "c" represent the major crystallographic direction of the particle.

The chemical analyses conducted on the external modified layer of the particles (e.g., Fig. S.3.2) demonstrate that phosphorus is absent (Fig. S.3.3; see also map shown in Fig. 12 of main text). The presence of Na in the spectrum is a good indicator of the chemical stability of the analyzed regions at the analytical conditions of our experiments.

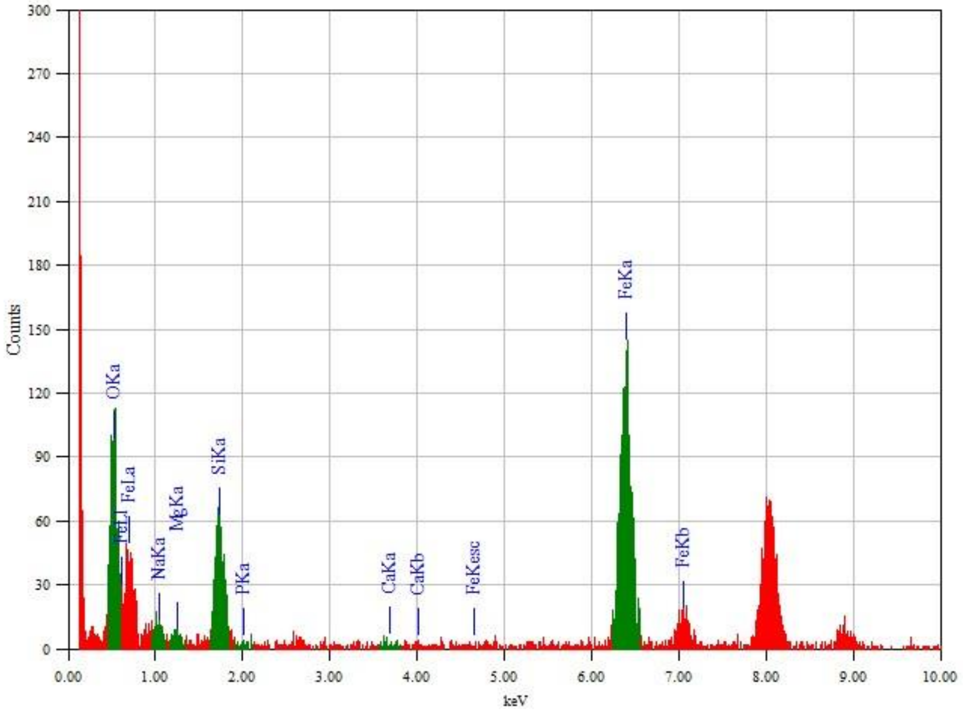

Fig. S.3.3 Example of a typical (raw) EDX spectrum collected on the external modified Fe-rich layer (shell plus nanoparticles) of amphibole particles extracted from AECs. The P signal is absent. The main peaks are highlighted in green. The large red peak at 8 keV is Cu (from the grid).

The Fe-rich layer, clusters, and nanoparticles are almost exclusively composed of Fe and O, and only rarely contain traces of other elements (Fig. S.3.4).

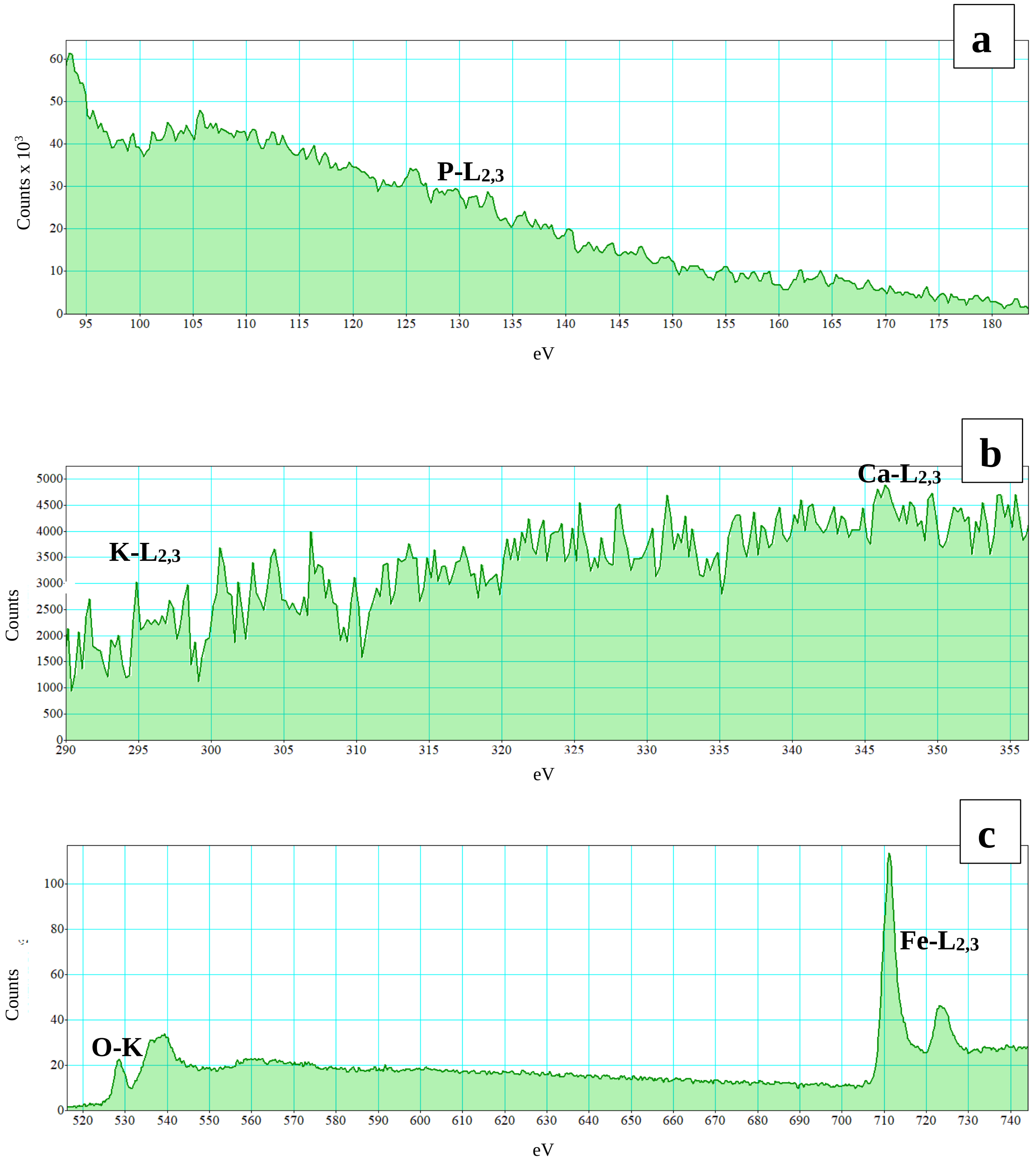

Fig. S.3.4 Raw background-subtracted high-loss EELS spectrum collected on a representative Fe-rich region **a)** section of the EELS spectrum documenting that the Si–$L_{2,3}$ (entire visible range) and P-$L_{2,3}$ (expected edge position labelled) edges and related peaks are absent; **b)** detail of the K–and Ca–$L_{2,3}$ edges (with the expected edge positions labelled) showing noise only and no distinguishable peak; **c)** detail of the well-defined O–K and Fe–$L_{2,3}$ edges.

The Si signal usually increases moving along profiles from the surface of the Fe-rich layer through the SiRA and to the boundaries of the amphibole particle (red arrow), see Fig. S.3.5.

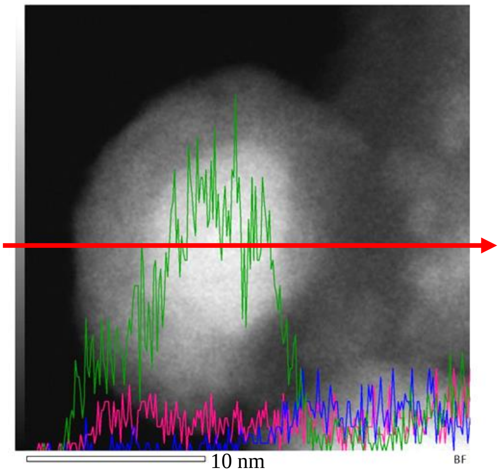

Fig. S.3.5 STEM-EDXS line profile (red arrow) of a Fe-rich nanoparticle within a Fe-rich layer that lies on the SiRA and is part of the amphibole boundary. While moving from left to right, the Fe signal (green line) drops from its peak in the Fe-rich nanoparticle, while the Si signal increases (blue line). The O is represented by the pink line.

S.4 Preliminary viability test on cell cultures

Our preliminary studies on the biological effects of the selected amphiboles were performed *in vitro* on alveolar epithelial cells (AEC), cell line A549 (American Type Culture Collection, USA, cell number ATCC CCL-185TM). These AECs were grown at standard growth conditions (5 % CO_2, 37 °C, 90 % relative humidity) in F-12K Nut mix medium (21127022 Gibco, USA) supplemented with 10 % HIFBS (F4135 Sigma, USA), 1 % L-glutamine (200 mM, M11-004 Gibco, USA), and 1 % Antibiotic-Antimycotic (100x, 15240 Gibco, USA). The cells were passaged twice a week using 0.25 % Trypsin-EDTA (T4049 Sigma, USA) for detachment. Amphibole particles were added to cell cultures at 50 µg/mL, and incubated for 48 hours. Cytotoxic effects of the amphiboles on the AECs were evaluated using the cell viability reagent PrestoBlue (A13261 ThermoFischer Scientific, USA). In order to perform the viability assay, the AECs were seeded one day prior to the experiment on 96-well microtiter plates at a density of 1.5×10^4 cells per well. For the treatment, the initial growth medium was replaced with serial dilutions of mineral suspensions in the growth medium at concentrations ranging from 0.2 to 100 µg/mL. The microtiter plates were incubated for 48 hours at standard conditions described above. After incubation, PrestoBlue reagent was added to each well (10 % v/v) and the cells were further incubated for 3 hours in a cell incubator (Steri-Cult 3307, Thermo Electron Corporation). The resulting fluorescence (λ_{ex}=560 nm / λ_{em}=590 nm) was measured using a FLUOstar Galaxy plate reader (BMG Lab Technologies, Germany). For the negative control, cells were grown in the culture medium in the absence of minerals, and their fluorescence values were taken as 100 % of cell viability. The tests were performed in triplicates.

Viability test showed a statistically not significant culture expansion. At the end of the 48-h exposure, the control cells reached confluence.

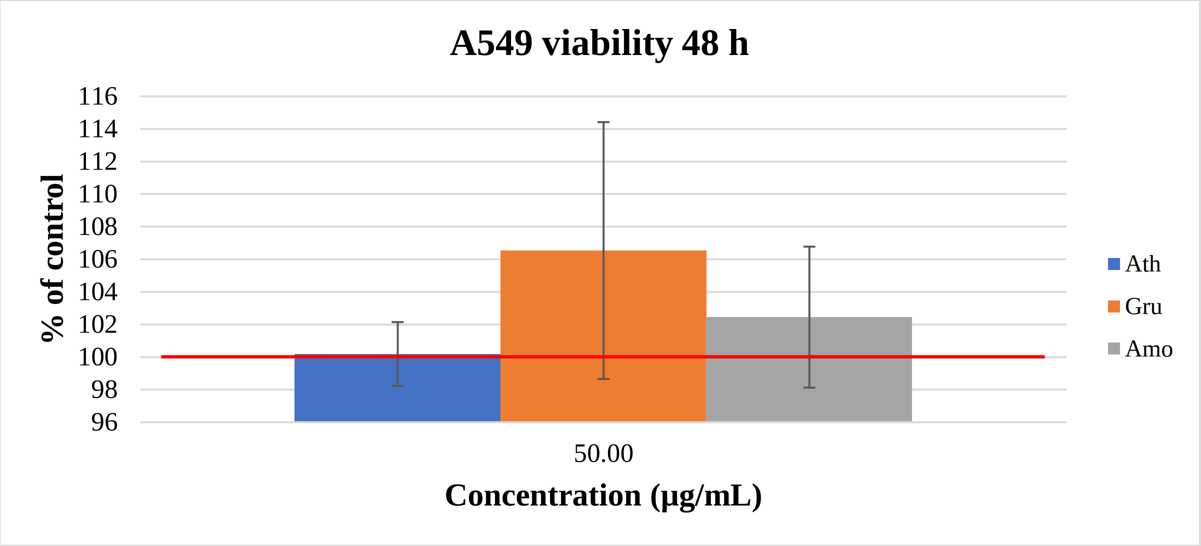

Fig. S.4.1 Cell viability (% of control) for the concentration (50 µg/L of different amphiboles used in our experiments. The red line highlights the control value (100%).

The apparent (statistically not significant) increase in viability may be a consequence of particle agglomeration[87] and may also be influenced by the additional surface area that long fiber bundles or particle agglomerates can provide to the cells, thus inducing cell growth.

S.5 Artefacts and stability test

In order to avoid misinterpretation of our results, all materials were tested for possible artefacts, which could arise during either the preparation steps or the direct observation under the S/TEM electron beam.

The possibility of artefact generation due to the use of media is mostly represented by cooling the media from 37 °C to room temperature, which can lead to precipitation of salts that cover the particle surfaces and other materials of interest. In our experiments the suspensions were always rinsed with warm deionized water (40 °C) before transfer onto the TEM grids to avoid salt formation. To simulate failure in this rinsing procedure we exposed the amosite particles to the media alone for the same experimental time as in the main experiment. Then, we transferred these particles from the suspension directly to the TEM grids without performing a rinsing step. This experiment generated a distinct thick amorphous salt-based covering on the amphibole particles as well as several salt crystals spread across the TEM grid, but the procedure did not affect the amphibole particle crystallinity (Fig. S.4.1).

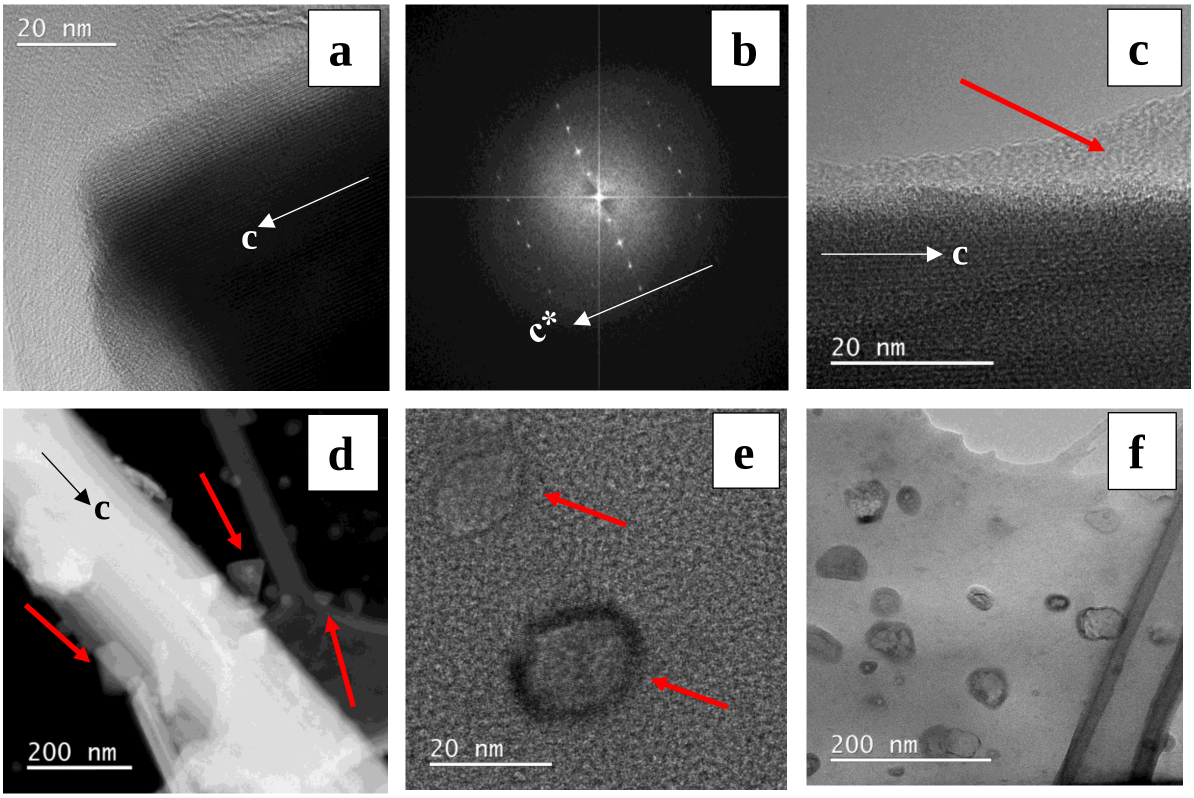

Fig. S.4.1 a) A highly crystalline amosite particle, covered by amorphous salts (HRTEM); **b)** Characteristic SAED pattern of the bulk amosite shown in **a)**; **c)** Irregular amorphous salt cover (red arrow) on a crystalline amosite particle (HRTEM); **d)** STEM-DF image of polygonal crystallized salts (red arrows) formed near an elongated particle; **e)** Highly crystalline salt crystals, highlighted by red arrows (TEM); **f)** Several rounded salt particles (TEM). The arrows labelled with "c" represent the major crystallographic direction of the particle.

Another process that can lead to artefact generation is through the electron beam. In order to assess the likelihood of inducing artefacts with the electron beam, we exposed different regions of amphibole crystals to the beam to test whether or not it was possible to generate a SiRA layer at the particle boundary under our experimental conditions. The exposure of the particle boundaries to the beam never generated amorphous material under the experimental conditions used. The only case in which we observed an actual morphological transformation and literally a movement of the material under the beam, was when we exposed an existing Si-rich amorphous portion of a particle to the beam. We believe that this may be related to the fact that this material was an entire rod made of amorphous Si and thus that the charging, combined with electron-heating, generated the morphological modification and movement of this peculiar portion of the material. The amorphous material was basically welded to the particle boundary (Fig. S.4.2). The particle crystallinity did not change during the process.

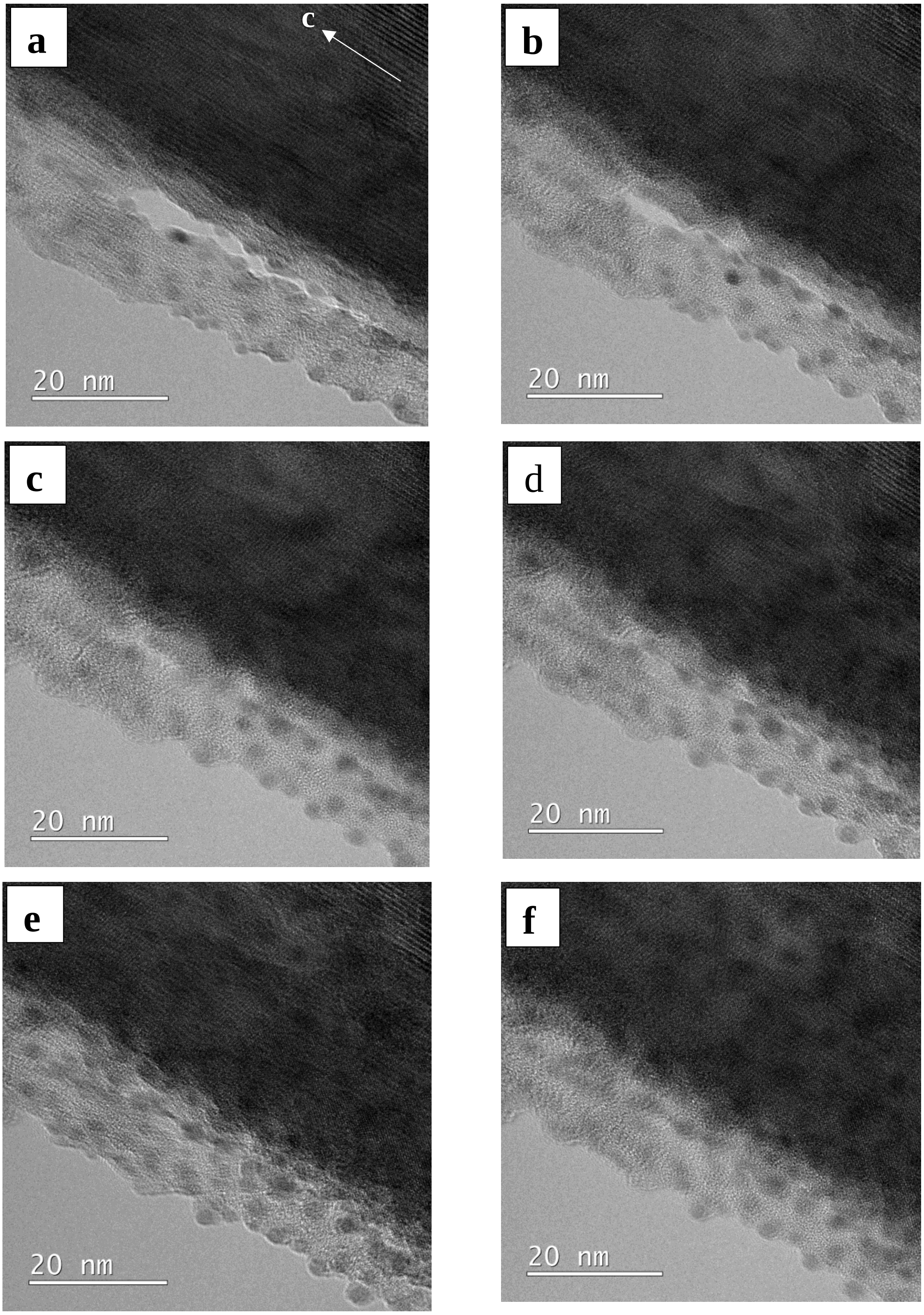

Fig. S.4.2 a) to f) Time series of intensive exposure to the electron beam of an amosite particle boundary. The images are recorded at 30s intervals (HRTEM). The arrow labelled with “c” represents the major crystallographic direction of the particle.

Supplementary information references

73. Rancourt, D.G., Ping, J.Y., Voigt-based methods for arbitrary-shape static hyperfine parameter distributions in Mössbauer spectroscopy. Nuclear Instruments and Methods in Physics Research B (NIMB), **58**, 85 - 97 (1991)
74. Della Ventura, G., Mottana, A., Parodi, G.C., Raudsepp, M., Monazite-huttonite solid-solutions from the vico volcanic complex, latium, Italy. Mineralogical Magazine, **60**(5), 751-758. (1996)
75. Della Ventura, G. The analysis of asbestos minerals using vibrational spectroscopies (FTIR, Raman): crystal-chemistry, identification and environmental applications. EMU Notes in Mineralogy, **18**, 135-169 (2017)
76. Rinaudo, C., Belluso, E., Gastaldi, D., Assessment of the use of Raman spectroscopy for the determination of amphibole asbestos. Mineralogical Magazine, **68**, 455-465 (2004)
77. Waeselmann, N., Schluter, J., Malcherek, T., Della Ventura, G., Oberti, R., Mihailova, B., Nondestructive determination of the amphibole crystal-chemical formulae by Raman spectroscopy: one step closer. Journal of Raman Spectroscopy, **51**(9), 1-19 (2019)
78. Redhammer, G.J., Roth, G., Crystal structure and Mossbauer spectroscopy of the synthetic amphibole potassic-ferri-ferrorichterite at 298 K and low temperatures (80–110 K). European Journal of Mineralogy, **14**, 105–114 (2002)
79. Iezzi, G., Della Ventura, G., Hawthorne, F.C., Pedrazzi, G., Robert, J.L., Novembre, D., The (Mg, Fe^{2+}) substitution in ferri-clinoholmquistite, $\square Li_2$ $(Mg,Fe^{2+})_3$ Fe^{3+}_2 Si_8 O_{22} $(OH)_2$. European Journal of Mineralogy, **17**, 733-740 (2005)
80. Della Ventura, G., Redhammer, G.J., Iezzi, G., Hawthorne, Papin, A., Robert, J.L., A Mössbauer and FTIR study of synthetic amphiboles along the magnesioriebeckite – ferri-clinoholmquistite join. Physics and Chemistry of Minerals, **32**, 103-113 (2005)
81. Della Ventura, G., Redhammer, G., Robert, J.L., Sergent, J., Iezzi, G., Cavallo, A., Crystal-chemistry of synthetic amphiboles along the join richterite - ferro-richterite: a combined spectroscopic (FTIR, Mössbauer), XRPD and microchemical study. Canadian Mineralogist, **54**, 97-114 (2016)
82. Hawthorne, F.C., Oberti, R., Harlow, G.E., Maresch, W.V., Martin, R.F., Schumacher, J.C., Welch, M.D., Nomenclature of the amphibole supergroup. American Mineralogist, **97**, 2031-2048 (2012)

83. Hawthorne, F.C., Della Ventura, G., Short-range order in amphiboles. in "Amphiboles: Crystal Chemistry, Occurrence and Health Issues", Hawthorne F.C. Oberti R. Ventura G. Della Mottana A., eds. Reviews in Mineralogy and Geochemistry, **67**, 173–222 (2007)
84. Linares, J., Regnard, J.R., Van Dang, N. (1983) Magnetic behaviour of grunerite from Mössbauer spectroscopy. J. Magnetism and magnetic materials, 31-34, 715-716.
85. Crawford, D., Electron microscopy applied to studies of the biological significance of defects in crocidolite asbestos. Journal of Microscopy, **120**, 181-192 (1980)
86. Hochella, Jr. M.F., Surface chemistry, structure, and reactivity of hazardous mineral dust. Reviews in Mineralogy, **28** (Guthrie G.D., Jr., Mossman B.T. editors), Health effects of mineral dusts, Chapter 8, 275-308 (1993)
87. Yao, S., Iezzi, G., Della Ventura, G., Bellatreccia, F., Petibois, C., Marcelli, A., Nazzari, M., Lazzarin, F., Di Gioacchino, M., Petrarca, C., Mineralogy and textures of riebeckite asbestos (crocidolite): The role of single versus agglomerated fibres in toxicological experiments. Journal of Hazardous Materials, 340, 472-485 (2017)
